# Design, Synthesis, Anti-Inflammatory Activity, DFT Modeling and Docking Study of New Ibuprofen Derivatives

**DOI:** 10.3390/ijms25063558

**Published:** 2024-03-21

**Authors:** Abbas M. Abbas, Hossam H. Nasrallah, Ahmed Aboelmagd, Safaa M. Kishk, W. Christopher Boyd, Haitham Kalil, Adel S. Orabi

**Affiliations:** 1 Chemistry Department, Faculty of Science, Suez Canal University, Ismailia 41522, Egypt; hossam.hamd@su.edu.eg (H.H.N.); ah_abuoelmaged@hotmail.com (A.A.); orabiadel@hotmail.com (A.S.O.); 2 Chemistry Department, Faculty of Dentistry, Sinai University, Kantara 41612, Egypt; 3 Medicinal Chemistry Department, Faculty of Pharmacy, Suez Canal University, Ismailia 41522, Egypt; safaa_keshk@pharm.suez.edu.eg; 4 Chemistry Department, College of Arts and Sciences, Cleveland State University, Cleveland, OH 44115, USA; w.c.boyd59@csuohio.edu

**Keywords:** ibuprofen, anti-inflammatory, acetylacetone, DFT, docking, complexes

## Abstract

A new ibuprofen derivative, (*E*)-2-(4-isobutylphenyl)-N′-(4-oxopentan-2-ylidene) propane hydrazide (IA), was synthesized, along with its metal complexes with Co, Cu, Ni, Gd, and Sm, to investigate their anti-inflammatory efficacy and COX-2 inhibition potential. Comprehensive characterization, including ^1^H NMR, MS, FTIR, UV–vis spectroscopy, and DFT analysis, were employed to determine the structural configurations, revealing unique motifs for Gd/Sm (capped square antiprismatic/tricapped trigonal prismatic) and Cu/Ni/Co (octahedral) complexes. Molecular docking with the COX-2 enzyme (PDB code: 5IKT) and pharmacokinetic assessments through SwissADME indicated that these compounds have superior binding energies and pharmacokinetic profiles, including BBB permeability and gastrointestinal absorption, compared to the traditional ibuprofen standalone. Their significantly lower IC50 values further suggest a higher efficacy as anti-inflammatory agents and COX-2 inhibitors. These research findings not only introduce promising ibuprofen derivatives for therapeutic applications but also set the stage for future validation and exploration of this new generation of ibuprofen compounds.

## 1. Introduction

Some biological features of ibuprofen, an anti-inflammatory, antipyretic, and analgesic compound, have proven helpful in the treatment of rheumatoid arthritis and other rheumatic disorders [1].

Ibuprofen and hydrazides of various carboxylic acids (both aliphatic and aromatic) reacted to produce oxadiazoles in the presence of phosphorousoxy chloride [2]. The condensation of 2-(4-isobutylphenyl)propane hydrazide (ibuprofen-hydrazide) with *N*,*N*-dimethylaminobenzaldehyde produced *N*′-(4-(dimethylamino)benzylidene)-2-(4-isobutylphenyl)propane hydrazide, which was utilized to create seven-membered ring compounds, and the condensation of ibuprofen-hydrazide with 4-chlorosalicylaldehyde yielded *N*′-(5-chloro-2-hydroxybenzylidene)-2-(4-isobutylphenyl)propane hydrazide. Different spectroscopy methods were employed to confirm all of these produced compound structures [3].

The acyl hydrazones were created using a conventional approach. Aromatic aldehydes were added to the ibuprofen hydrazide solution in dry ethanol. The reaction mixtures were refluxed until the reaction was complete. Using a rotary evaporator, after the solution had cooled to ambient temperature, the solvent was removed. The residue was purified using dichloromethane-methanol as the eluent on a silica gel column. For acyl hydrazones, FT-IR and physical data have been calculated [4,5].

An Au(I)–ibuprofen complex [Au(ibup)(CN)] has been synthesized and characterized. The complex’s antibacterial action against Gram-negative and Gram-positive microorganisms was discovered in vitro using an antibiotic-sensitive profile [6].

Complexes of the vanadyl ion (VO^2+^) with tolmetin, naproxen, and ibuprofen were acquired from methanolic solutions under nitrogen, and have the general formulas [VO(Tol)_2_], [VO(Nap)_2_]·5CH_3_OH, and [VO(Ibu)_2_]·5CH_3_OH. The biological activities of the complexes were compared with those of the vanadyl cation after culturing two types of osteoblast-like cells. The activity of the complexes varied depending on the cell type and concentration, whereas their parent drugs had no effect [7].

According to Raman’s study of ibuprofen–metal complexes, Co(II) produces monodentate complexes, whereas Zn(II) adopts bidentate coordination. The synthesis of a stable 2:1 ligand/metal complex appears difficult at ppm concentration, on the basis of spectra of metal complexes using surface-enhanced Raman spectroscopy (SERS), but more plausible is the creation of a 1:1 adduct on the carboxylic group through bidentate binding. Water molecule complexation is linked to the total coordination shell of a metal [8]. The silver–ibuprofen complex (Ag–ibu) was formed at a 1:1 ratio. The Ag–ibu complex minimum inhibitory concentration (MIC) values vary from 6.25 to 12.5 L g/mL and have antimicrobial effects against both Gram-positive and Gram-negative bacteria [9].

Our main objective is to produce several new derivatives from ibuprofen according to prior research demonstrating the biological activity of such derivatives, predicated on the premise that specific modifications could result in compounds with therapeutic promise that have improved anti-inflammatory action and lower negative effects. First, acetylacetone and ibuprofen hydrazide were combined to form a novel molecule that was then combined with different metal ions. The ligands and their compounds were characterized using several approaches. Experiments and in silico analyses were performed to determine the physicochemical properties. To estimate the biological behavior of the compounds theoretically, docking studies were performed on the COX-2 protein. The pharmacokinetics of IA and its cobalt complex were modeled using the SwissADME online web tool. To investigate the derivatives and their complexes in vitro, Western blot and ELISA analyses against COX-2 were performed. Theoretical and experimental data suggest that these new compounds may have potential as drugs. In vivo experiments with an animal model will be part of further studies.

## 2. Results and Discussion

### 2.1. IA Description

The purity and reaction efficacy of the new compounds were evaluated using TLC using the suitable elution mixture solution (90%:10% chloroform/ethanol). The experimental section describes how ibuprofen ester (EI) and its hydrazide (HI) were prepared. Ibuprofen hydrazide and acetylacetone were condensed to form the ligand IA (C_18_H_26_N_2_O_2_), which was produced with a yield of 75%.

IA is a crystalline substance with a canary-yellow color and melting point of 159 °C. The solubility of IA in different solvents was investigated, and it was found to be soluble in alcohols, DMSO, and DMF. The actual chemical structure was confirmed by the elemental analysis of IA, which yielded the following results: calculated (found): H% = 7.46 (7.51), N% = 8.63 (8.55), and C% = 74.05 (74.10) (Table 1).

The IA ^1^HNMR spectra indicated an associated singlet peak for the (N-N-H) proton at 9.95 ppm and the aromatic protons showed at 7.24–7.05 (4H, m, ArH). The number of protons in each group corresponded to all the obtained peaks and integration. Table S1 shows the ^1^HNMR spectral information for HI and IA. Technically, the research on ibuprofen’s conformational behavior in different solvents using NMR techniques illustrates the molecule’s structural adaptability. In chloroform, 2D NOE spectroscopy identified ibuprofen’s preferred conformations, offering insights into how these structures influence crystallization processes [10]. High-pressure NMR spectroscopy revealed ibuprofen’s dominant conformers in supercritical carbon dioxide, aligning with molecular dynamics simulations [11]. These studies underscore NMR’s potency in unraveling molecular conformations, critical for drug formulation and understanding pharmaceutical compounds’ physicochemical properties [10,11]. However, our study has primarily employed NMR spectroscopy to confirm the structural integrity of the new ibuprofen derivative without deeply exploring the range of potential conformations. Our results corroborate the efficacy of NMR confirming the successful synthesis of our new ibuprofen derivative, IA, aligning with the direct approach of using NMR to understand molecular configurations (Figure S1).

The chromatogram (Figure S2) with a single peak demonstrated the purity of the IA and HI ligands, and the ligand formula was supported by the mass spectrum. The molecular ion peak for HI and IA was determined using the molecular weights at *m*/*z* = 221.11 (11.02%) and 302.51 (24.02%). The stepwise fragmentation of the ligands was established and confirmed (Schemes S1 and S2). The collected fragments and the presented results demonstrate the accuracy of molecular formula of IA and HI.

The stretching vibration of the hydroxyl group is responsible for the large, broad band at 3416 cm^−1^ observed in the FT-IR spectrum of ibuprofen, and the band’s broadness can be ascribed to intramolecular hydrogen bonding. C=O stretching is associated with the band at 1709 cm^−l^. Figures S3 and S4 depict the FT-IR spectra of the IA. The stretching vibration of the O-H group was associated with a strong broadband at 3419 cm^−1^. These bands’ broadening could be due to an intramolecular hydrogen bond [12].

A sharp and strong band at 3413 cm^−1^ was observed, corresponding to the stretching vibration of the NH group (υNH) [13]. The bands at 1725 and 1660 cm-1 can be designated as (υC=O) from the C=O group’s stretching vibration [14], and the occurrence of the band at 1611 cm^−1^ is proposed to result from the azomethine group stretching vibration (υC=N) [15]. The NH_2_ vibration band is absent, and the occurrence of a C=N band serves as confirmation of the formation of IA (Table 2).

The UV–vis spectrum of IA exhibited three bands at 257, 264, and 272 nm (ε = 10.34, 11.27, 10.59 × 10^4^ M^−1^ cm^−1^), as shown in Figure S5. In methanol solvent, these bands displayed a blue shift, showing the n→π* and π→π* electronic transitions.

The thermal decomposition of the ligand was investigated using TG and DTA. The temperature range covered by the thermal evaluation was 200–600 °C, which occurred in a N_2_ environment with a heating rate of 10 °C/min. The thermal decomposition of IA shows that mass reduction occurs gradually. DTA data and TGA from the IA are displayed in Figure S6.

Two decomposition phases are present in the IA thermogram at 261–321 °C and 443–473 °C, with midpoints of 306 and 458 °C and mass losses of 95% and 5%, respectively, without any residue (650 °C). Δ*H* values of −0.401 and −0.0147 kJ/g, respectively, indicate that all steps exhibit exothermic behavior at 355 and 460 °C [16]. The endothermic peak in the DTA data confirms the melting point of IA at 130 °C (Δ*H* = 0.017 kJ/g), with no weight loss (Table S2). The purity and efficacy of the IA as a coordination complex synthon for metals in the *d*- and *f*-blocks have also been demonstrated by extensive spectroscopic and thermal studies.

### 2.2. Complex Characterization

The new complexes [Cu(IA)(H_2_O)Cl_2_]·6H_2_O, [Ni(IA)(H_2_O)_2_Cl]Cl·H_2_O, [Co(IA)(H_2_O)Cl_2_]·2H_2_O, [Gd(IA)_2_(NO_3_)_2_(H_2_O)]NO_3_·2H_2_O and [Sm(IA)_2_(NO_3_)_2_]NO_3_·3H_2_O were formed as a result of the interaction between IA with Cu(II), Ni(II), Co(II), Gd(III), and Sm(III) ions. Each complex is crystalline and soluble in DMF or DMSO, excluding the Cu, Ni, and Co complexes, which are soluble in DMF solvent after heating and have melting points above 280 °C. The electrical conductivity of complexes was measured in DMSO (0.001 M), indicating the electrolytic feature of the compounds, with conduct similar to that of the 1:1 electrolyte, except for Cu and Co complexes being non-electrolytes. Table 1 displays the physical properties of the IA and its metal complexes as well as the conductivity, CHN, and M% values. The M% content was determined using two analytical procedures: EDTA titration and thermogravimetry. These results match those of previous formulas [17,18].

#### 2.2.1. FTIR Spectra

**Figure 1:**
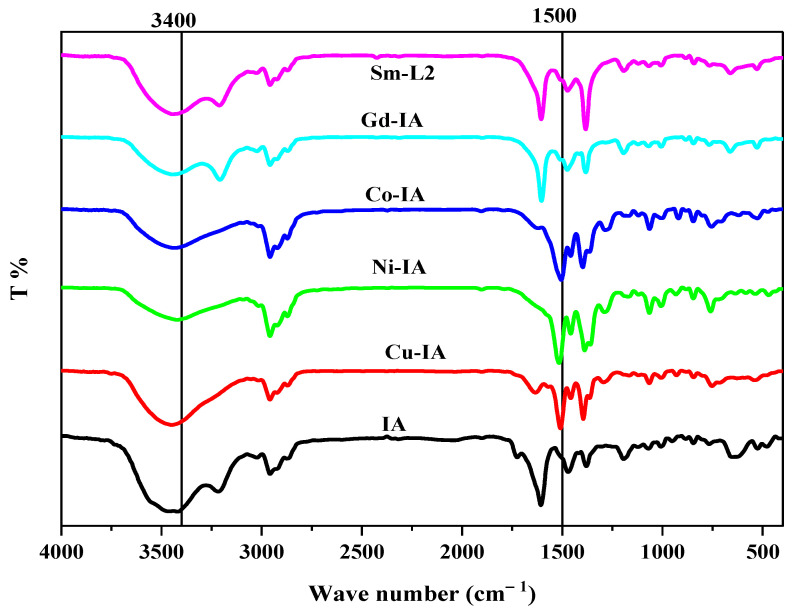
FTIR spectrum of IA and its complexes.

By comparing the IR spectra of the IA with its complexes, the coordination sites expected to be important to complexation were identified. Peak positions and/or intensities will likely shift as a result of chelation, as explained in Table 2, Figure 1 and Figures S7–S11.

The strong, broad band at 3800–2900 cm^−1^ could be attributed to the stretching vibrations of H_2_O, O-H, and N-H. This band was present in every complex, confirming that the water was crystalline or coordinated, due to the υ(OH) and υ(NH) of IA [19,20]. The C=O group showed two peaks for IA at 1725 and 1660 and one peak of complexes at 1641, 1608, 1612, 1612, and 1617 cm^−1^, showing that this group is ligated during the chelation of Cu(II), Ni(II), Co(II), Gd(III), and Sm(III) [21,22,23]. The bands shown for IA, Co–IA, Cu–IA, Ni–IA, Gd–IA, and Sm–IA complexes at 1611, 1508, 1528, 1510, 1528, and 1518 cm^−1^ could be represented by the azomethine group stretching vibration, where the changes in the frequencies confirm the complexation. In Gd(III) and Sm(III) complexes, the sharp band at 1384 cm^−1^ is characterized as υ(NO_3_) and either reflects the nitrate group’s ionization activities or acts as a monodentate binding site [24,25].

The M-O stretching band appeared at 636, 622, 617, 651, and 680 cm^−1^, while the M-N band appeared at 526, 540, 521, 531, and 516 cm^−1^ for Co–IA, Cu–IA, Ni–IA, Gd–IA, and Sm–IA, respectively.

#### 2.2.2. Thermal Analysis

**Figure 2:**
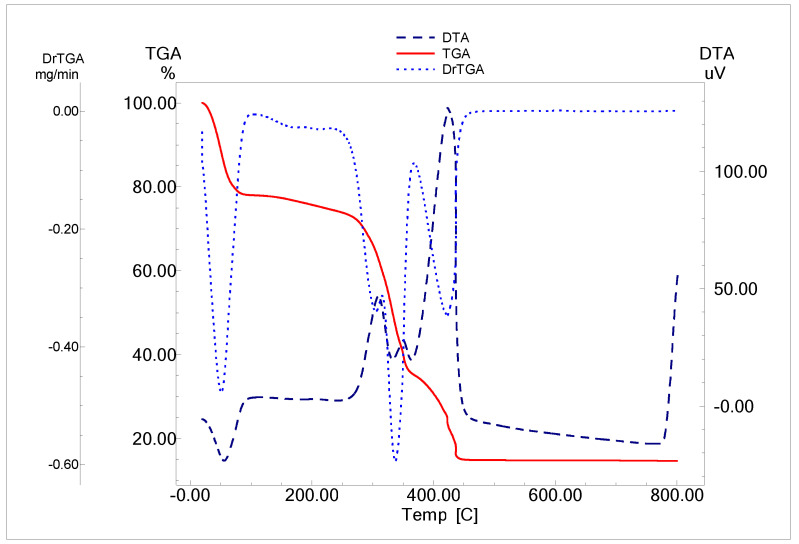
The [Cu(IA)Cl_2_.H_2_O]6H_2_O complex TGA/TG and DTA curves.

Thermal analysis (TG/DTG) and differential thermal analysis (DTA) were used to examine the thermal stability of the complexes. Table 3 and Figure 2 and Figures S12–S15 display the decomposition products, temperature ranges, stages of decomposition, and the estimated and actual weight loss of complexes formed through IA.

The first decomposition step involves the loss of water of crystallization. [Cu(IA)Cl_2_(H_2_O)]·6H_2_O, [Ni(IA)(H_2_O)_2_Cl]Cl·H_2_O, [Co(IA)(H_2_O)Cl_2_]·2H_2_O, [Gd(IA)_2_(NO_3_)_2_(H_2_O)]NO_3_·2H_2_O and [Sm(IA)_2_(NO_3_)_2_]NO_3_·3H_2_O underwent dehydration at 49, 49, 53, 38 and 98 °C, respectively. This process resulted in mass losses of 19.70, 3.65, 7.35, 4.21, and 5.45 (calcd. 19.20, 3.71, 7.41, 3.59, and 5.34), concurrent with endothermic DTA peaks at 56, 86, 46, 48, and 97 °C, respectively.

In addition to IA decomposition, the second phase of decomposition shows coordinated loss of water and/or HCl and a broad DTG band at 304, 341, 271, 280, and 279 °C for Cu–IA, Ni–IA, Co–IA, Gd–IA, and Sm–IA complexes. The measured mass loss through this step was (found/calculated %) 15.14/15.16, 50.46/50.01, 46.62/46.56, 33.64/33.65 and 53.06/53.11, respectively. DTA thermogram appeared as exothermic DTA bands with maxima at 308, 352, 273, 352, and 340 °C, for Cu–IA, Ni–IA, Co–IA, Gd–IA, and Sm–IA.

The remaining ligand decomposition and/or HNO_3_ are indicated in the third decomposition stage, which was initiated at 338, 420, and 460 °C for Cu–IA, Gd–IA, and Sm–IA. The lost mass was (found/computed %) 30.84/30.81, 15.82/15.92 and 6.62/6.63, respectively. Cu, Gd, and Sm DTA thermograms revealed this step as an exothermic DTA band at 350, 424, and 466 °C.

The final decomposition occurred at 423, 353, 393, 497, 548, 590, and 660 °C for Cu, Co Ni, and Sm complexes. The lost mass was (found/computed %) 20.75/20.56, 30.62/30.61, 30.50/30.52, 9.97/9.98 and 0.39/0.38. Cu, Ni, Co, Gd, and Sm DTA thermograms showed exothermic DTA bands at 423, 434, 400, 403, 351, 499, and 512 °C. The computed data and suggested formulas of CuO, NiO, CoO, Gd_2_O_3_, and Sm_2_O_3_ agree with the residue %, with lost mass (found/computed %) 14.50/14.15, 12.41/15.37, 18.99/15.40, 33.25/36.17 and 30.01/35.03. For each step that displayed exothermic behavior, Table S3 displays the enthalpy change and peak temperature.

#### 2.2.3. Kinetics and Thermodynamic Parameters

The TGA and DTG thermograms were employed to compute the activation energy (*E*_a_), pre-exponential factor (Z) and order (*n*) of the various decomposition processes to investigate the influence of ligand structural characteristics on complex thermal analysis [26,27]. Figures S16–S20 and Table 4 illustrate some complex linearization curves that can be produced with the Coats–Redfern equation. The intensity and appearance of the degradation were taken into consideration when choosing the breakdown processes. Analysis of the kinetic parameters (ΔG*, ΔH*, and ΔS*) for the degradation stage gives interesting results.

Among other factors, the reaction’s temperature affects the collision frequency. The particles’ average speed and kinetic energy also increases as the temperature does. Activation energy is necessary for reactant molecules to get close to each other, overcome repulsion forces, and initiate bond breaking. We can summarize our results using the collision theory as a foundation:

1. The variations in ΔS* caused by the thermal degradation reactions indicated the disorder’s decreases, except the final stage of the complex [Cu(IA)(H_2_O)Cl_2_]6H_2_O. Negative values signify slower than usual reactions because the structure of the activated complexes is more ordered than that of the reactants.

2. The Cu complex has the largest activation energy (231.38 J/mol) and the largest *Z* for the IA decomposition (2.87 × 10^17^ s^−1^).

3. The selected decomposition steps show exothermic behavior, indicated by negative ΔH*.

4. In all complexes, Δ*G*^‡^ has a positive value and has demonstrated non-spontaneous behavior, except for Cu and Gd, which have negative values and displayed in Table 4.

5. The reactivity of complexes in thermal decomposition processes is represented by the activation energy value.

#### 2.2.4. Magnetic Moments and UV–Vis Spectroscopy

**Figure 3:**
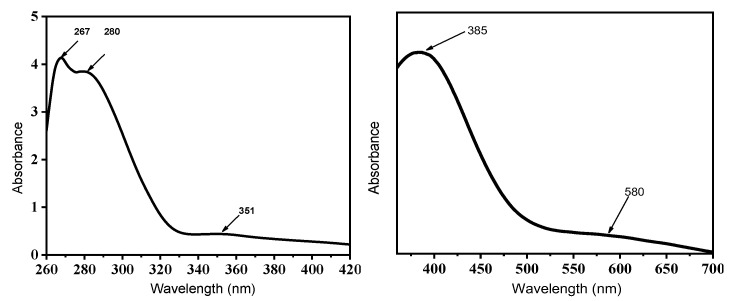
The Cu–IA UV–vis spectra.

The complexes’ spectral studies (in DMSO solution and Nujol mull suspension), also with their assignments, are shown in Figure 3 and Figures S21–S24 and collected in Table S4. The electronic transitions of IA shown at 257, 264, and 272 nm are due to π→π* and n→π* transitions, which submit a blue shift (hypochromic) accompanied by hyperchromic or hypochromic shifts after complexation, indicating complex formation [28].

The Cu–IA and Ni–IA magnetic moment values (*µ*_eff_) were 2.07 and 2.46 *µ*_B_, respectively, which indicate a distorted octahedral structure and could be linked to the outer complex formation (sp^3^d^2^ configuration). The electronic transition bands of Cu–IA appear at 267, 280, 351, 385, and 580, assigned for (n→π* and t_2g_→e_g_); meanwhile, in the electronic spectra of Ni–IA, bands appear at 276, 290, 402, and 620, assigned for (n→π*, ^3^A_2g_→^3^T_1g_(P) and ^3^A_2g_→^1^E_g_), and are tabulated in Table S4.

The Co–IA magnetic moment was 4.86 *µ*_B_, which was indicative of octahedral geometry with a high-spin ground state [29]. The electronic spectrum displays bands at 298 and 347 assigned for n→π* and _4_T_1g_(F)→_4_T_2g_(P) while the broad band with three maxima at 520, 558, and 603 can be assigned for _4_T_1g_(F)→_4_A_2g_(F) transitions, with the fine splitting possibly due to Jahn–Teller distortion effects [30].

The magnetic moment estimated for Sm–IA was 2.29 *µ*_B_, indicating the formation of a low-spin complex. Finally, the Gd–IA magnetic moment was 8.89 *µ*_B_, showing a greater contribution from *f*-electron spin and identifying the complex as highly paramagnetic (*f*^7^). Results are in agreement with earlier work [31]. Capped square antiprismatic/tricapped trigonal prismatic or bicapped trigonal prismatic/square antiprismatic geometries are the most likely structures for these lanthanide complexes.

The Gd–IA and Sm–IA compounds’ UV–visible spectra showed absorption bands at 275, 295, 311, and 273, 293, 310 nm as n→π* and CT transition [31,32].

The complexes were demonstrated by the difference in through bands between the ligand and the complexes. The metal bands are covered by high-intensity ligand or charge transfer bands. 10*Dq* values for Co, Cu, and Ni complexes are shown in Table S4 [33,34].

**Scheme 1:**
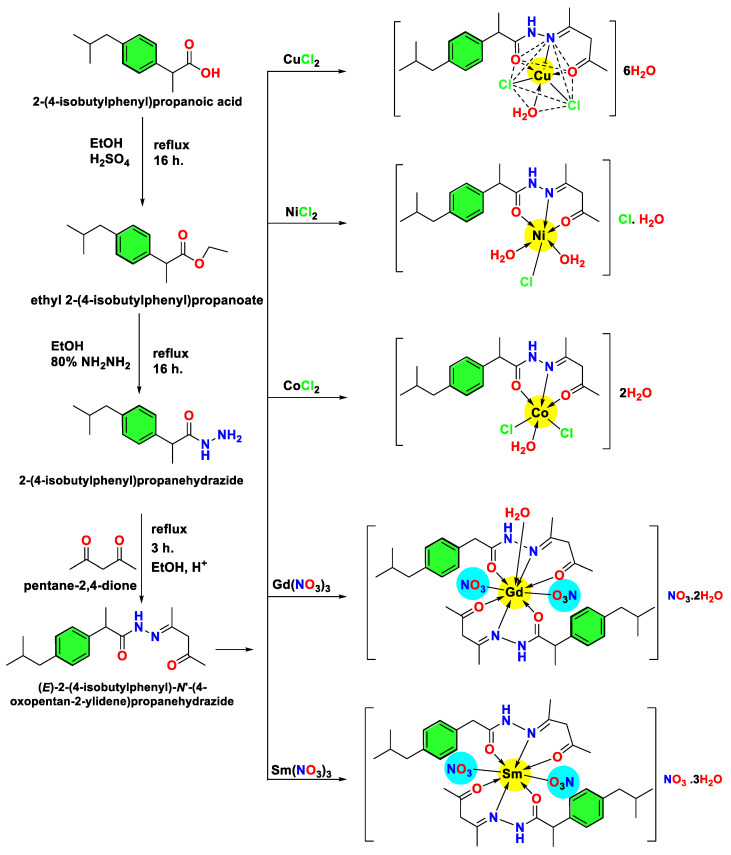
The expected structures of M(II)–IA or M(III)–IA complexes.

The structure and spatial arrangement of the complexes could be suggested based on molar conductivity, elemental analysis, magnetic properties, thermal analysis, FTIR and UV–visible spectra, as depicted in Scheme 1.

### 2.3. Computational Chemistry

The biological availability of drug candidates can be predicted via computational modeling of electron density and hydrophilicity vs. lipophilicity. The docking studies, which estimate the energies of interaction between target biomolecules like proteins and therapeutic potential chemicals, can help to explain a biological process. Docking studies were utilized in this section to investigate the interactions of ibuprofen, IA, and its metal complexes with the COX-2 enzyme. DFT calculations were used to determine the surface properties, electron density, and frontier molecular orbitals of these compounds (Section 2.4). The ideal target for the compound can also be predicted using the Swiss Target Prediction online tool. It was found that the ligand and Co–IA complex had a high rate of absorption by the GIT and passive GI tract permeability, when the potential of ibuprofen was calculated using the BOILED-Egg model (Section 2.5).

#### 2.3.1. The IA Ligand Surface Properties

**Figure 4:**
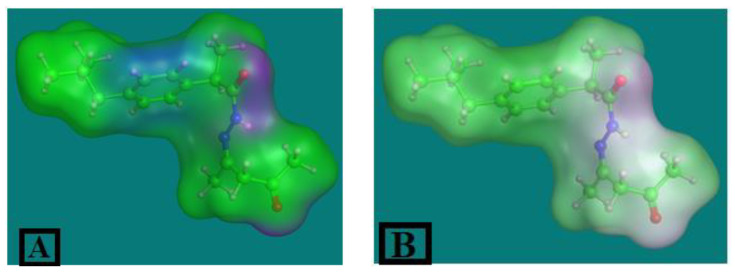
The IA surface properties. (**A**) Active lone pair map; (**B**) hydrophilic and lipophilic map.

The most advantageous properties essential for pharmaceutical manufacturing are related to the surface characteristics of IA. The interaction between living cells and the drug demonstrated the presence of a lipophilic property and an active lone pair. The active lone pair map was displayed in three colors: violet for H-bonding, green for hydrophobic, and blue for mild polarity. For IA, the H-bonding capacity was centered in three locations. Different colors were used to display the lipophilic map, with violet indicating hydrophilia, white showing neutrality, and green indicating lipophilia [35,36]. The lipophilic maps of IA are shown in Figure 4.

#### 2.3.2. The DFT Calculation for Ibuprofen and IA

Investigations into the MM2, DFT calculations, HOMO, and LUMO energies were performed on IA and ibuprofen. By using the MMFF94x force field parameter, each molecule undergoes energy minimization. Self-consistent field (SCF) calculations, also known as the Hartree–Fock method, were employed to run the simulation. Hamiltonians PM3 and RHF were used along with MOPAC (limited Hartree–Fock technique). 

The HOMO and LUMO orbitals provide information about the target orbitals, which are a part of the excitation–relaxation path of electronic transition. It was demonstrated that moving an electron from one level to another requires less energy due to the significant difference in the number of occupied and unoccupied orbitals. The drug behavior is determined by the polarity of ibuprofen (1.94 D).

**Figure 5:**
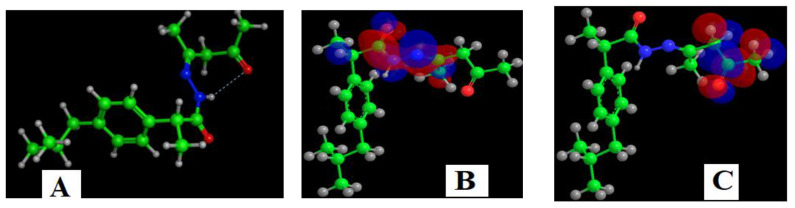
DFT modeling of the IA ligand. (**A**) 3D view, (**B**) HOMO, and (**C**) LUMO.

The dipole moment value (2.71 D) of IA is consistent with the requirements for the novel drug. The various molecular orbitals suggest the possibility of a large number of conformations that may have an interaction with the receptor, increasing the efficacy of the drug [36,37,38]. The results are shown in Figure 5 and Figure S25 and Table S5. The frontier molecular orbitals of IA (HOMO and LUMO) provide insight into the ability of IA to bind to transition metals and lanthanides as a ligand. In the HOMO, the high π-type electron density at the hydrazido oxygen and nitrogen suggest that IA can serve as a π-donor ligand, while the fact that the isolated carbonyl oxygen is not part of the HOMO suggests that this oxygen will more likely bind to a metal in a σ-donor fashion. In contrast, the LUMO of IA has considerable contribution from a C–O π-antibonding interaction. This suggests that IA may be able to exhibit some π-acceptor behavior in its bonding to metals, at least to Co(II), Ni(II), and Cu(II); such bonding is unlikely, however, in Sm(III) and Gd(III), which lack valence *d* electrons and whose valence 4*f* orbitals will not engage in strong covalent bonding.

We used DFT to determine the hardness and softness of the compounds. As a result, it was determined that:

1. The key equations describing the transition between ground states provide a robust framework for defining local, global, and non-local hardness and softness functions.
2. Under chemical potential, it has been shown that interactions between two systems evolve towards a state of maximum hardness. Additionally, both soft–soft and hard–hard interactions are observed to be preferentially established.
3. Lastly, it has been demonstrated that a system’s ground-state energy tends to decrease as its hardness increases, at least to a significant extent. These general principles could be instrumental in understanding the behavior of molecules in general and in discerning their reactions with various chemical types.

### 2.4. Molecular Docking Analyses

#### 2.4.1. Ibuprofen Docking with COX2 (PDB Code: 5IKT)

**Figure 6:**
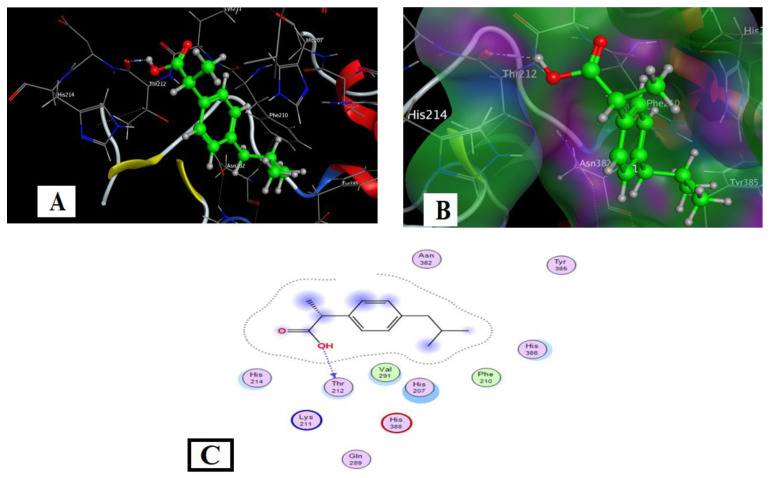
Ibuprofen’s docking with *Cox2*: (**A**) 3D view; (**B**) the surface characteristics (hydrophilic (violet-colored), lipophilic (green-colored), and neutral (white-colored) sites, and neutral sites (white color)); (**C**) 2D diagram.

Ibuprofen binds to the target protein via O(14) with the amino acid THR212. Hydrophobic interactions and hydrogen bonds were the most typical forms of binding bonds. The binding energy of the drug was found to be −5.33 kcal/mol, as showed in Table 5 and Figure 6.

The obtained model suggests that ibuprofen forms a single bond with the 5IKT protein via a hydrogen bond with THR 212 (H-donor, 2.85 Å) and demonstrates a binding energy of −2.9 kcal/mol. Similarly, the IA ligand forms a hydrogen bond with TYR 385 (H-donor, 3.05 Å), resulting in a binding energy of −0.7 kcal/mol. The preferred pose of the Co–IA complex interacts with 5IKT through three bonds with HIS214, HIS207, and HIS388, characterized by a hydrogen acceptor (HAc) interaction (3.19 Å, −5.2 kcal/mol), and two pi-cation interactions (3.78 Å, −0.6 kcal/mol; 3.70 Å, 0 kcal/mol), respectively. The Cu–IA complex forms two bonds with the 5IKT protein involving SER 451 and TYR385, marked by an HAc interaction (3.47 Å, −0.9 kcal/mol) and a pi-cation interaction (4.6 Å, −0.6 kcal/mol). The Ni–IA complex establishes three bonds with PHE 210, ASN382, and HIS 386 (H-donor, 2.73 Å, −2.2 kcal/mol; H-donor, 3.19 Å, −1.3 kcal/mol; pi-cation interaction, 4.6 Å, −0.6 kcal/mol). For the Gd–IA and Sm–IA complexes, interactions with 5IKT are mediated by HIS 386 (pi-cation interaction, 4.42 Å, −0.9 kcal/mol) for Gd–IA and by HIS 386 and HIS 207 (pi-cation interaction, 4.33 Å, −1 kcal/mol; pi-cation interaction, 4.86 Å, −0.7 kcal/mol) for Sm–IA. Overall, the total binding energy is influenced not only by its magnitude but also by the nature of the interactions, as observed in IA and its complexes.

#### 2.4.2. IA Docking of with COX2

**Figure 7:**
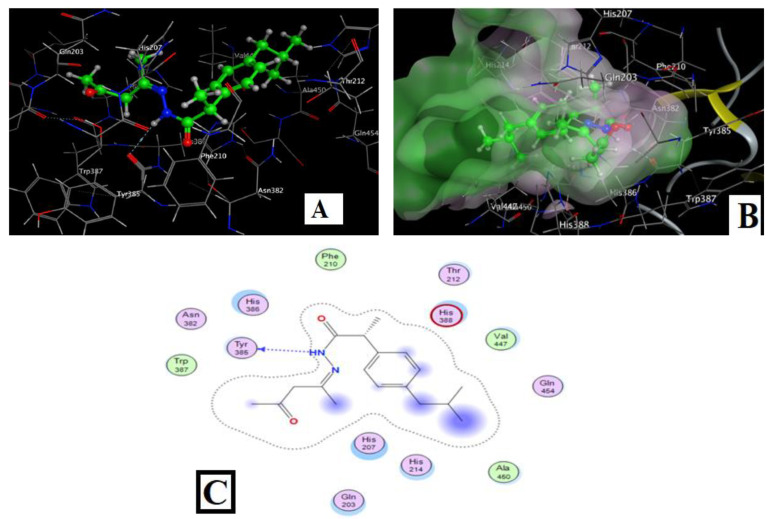
The docking between IA with Cox2: (**A**) 3D view; (**B**) the surface properties; (**C**) 2D view.

The obtained result (Table 5 and Figure 7) showed the docking between IA [N(14) of amide group(N-H)] and the COX2 protein [TYR 385] by H-bond. The binding energy (−6.48 kcal/mol) of the binding conformation was found to be superior to that of ibuprofen. The docking results revealed that the hydrazide group of IA binds to COX-2 in a manner analogous to the carboxylic group in ibuprofen. However, the distinctive nature of the hydrazide interaction contributes significantly to the enhanced anti-inflammatory activity. The complexes, comprising hydrazide, nitrate groups, chlorine atoms, and metal ions, exhibited improved connectivity with COX-2 compared to both the IA ligand and ibuprofen. Generally, the augmentation in receptor affinity observed in ibuprofen derivatives is attributed to alterations in their molecular composition, thereby elevating their potential for protein binding. These modifications have led to the development of more potent COX-2 inhibitors with significantly enhanced in vitro activity.

#### 2.4.3. Molecular Docking of the Complexes

**Figure 8:**
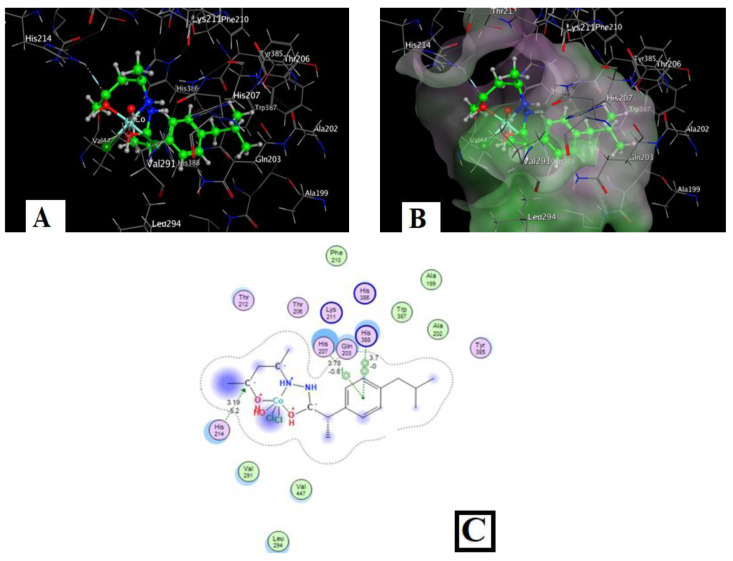
The docking of Co–IA with *Cox2*: (**A**) 3D view; (**B**) the surface features; (**C**) 2D view.

The interaction sites of all complexes are greater than IA and ibuprofen, except for Gd–IA, which is similar to the free IA but has more interaction sites than ibuprofen. The total binding energies for Co–IA, Cu–IA, Ni–IA, Gd–IA, and Sm–IA were −6.65, −7.45, −8.16, −7.41, and −9.14 kcal/mol, respectively, which are all more negative than both ibuprofen (−5.33 kcal/mol) and IA (−6.48 kcal/mol). The results are shown in Table 5 and Figure 8 and Figures S25–S29.

**Figure 9:**
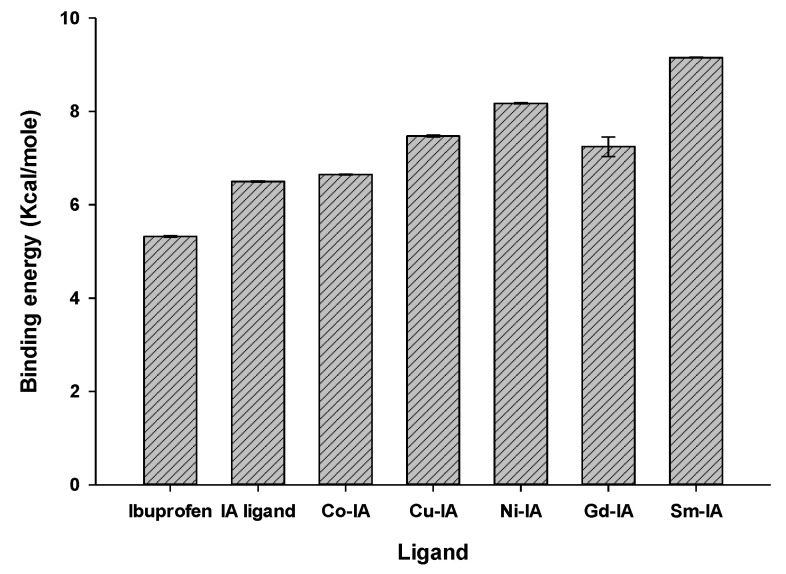
The binding energies of ibuprofen, IA, and its complexes.

The complexes contained more interaction sites and more negative binding energies with COX2 than ibuprofen and IA, with the exception of Gd–IA, which has one site (Figure 9). These theoretical predictions for the new compounds are promising, and we therefore expect them to have a biological effect in cell studies.

### 2.5. In Silico Studies

#### 2.5.1. Prediction of Target–Ligand Interactions

**Figure 10:**
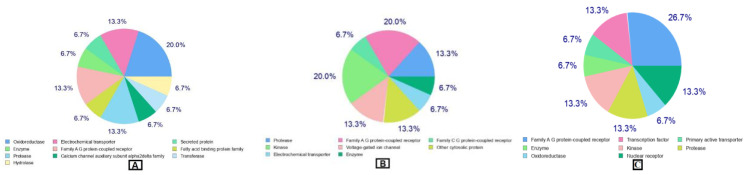
Target prediction of the proposed compounds using the Swiss Target Prediction Webtool. (**A**) Ibuprofen drug; (**B**) ligand IA; (**C**) Co–IA complex.

Using the Swiss Target Prediction website tool, we can predict the compound’s best target, and the model suggests, as shown in Figure 10, that ibuprofen may have an oxidoreductase receptor as a target, with a prospect of 20%, a family A G protein-coupled receptor, electrochemical transporter, and protease, with a probability of 13.3%. Other receptors were enzymes, families of fatty acid-binding proteins and alpha2delta calcium channel auxiliary subunits, hydrolase, secreted protein, and transferase, each with a probability of 6.7%.

IA was found to display inhibitory action to a kinase and family A G protein-coupled receptor, with a probability of 20%, while the voltage-gated ion channel, protease, and other cytosolic protein inhibition each have a probability of 13.3%. Other receptors were enzymes, electrochemical transporters, and family C G protein-coupled receptors, each with a prospect of 6.7%. Co–IA may possess inhibitory action for a family A G protein-coupled receptor with a prospect of 26.7%, while kinase, protease, nuclear receptor, and transcription factor inhibitory action each have a prospect of 13.3%. Other possible receptors were enzymes, oxidoreductase, and primary active transporters, each with a probability of 6.7%.

From the expected process, there is an increase in A G protein-coupled receptor for IA and Co–IA (20 and 26.7%), as contrasted with ibuprofen (13.3%) and appearance kinase receptor, with prospects of 20 and 13.3% for IA and Co–IA.

#### 2.5.2. Bioavailability Prediction

A molecule’s behavior in a living organism is affected by many factors, including bioactivity, transport properties, protein interactions, and other forms of reactivity. The SwissADME online web tool was modified to incorporate the physicochemical characteristics of the ibuprofen, IA, and Co–IA complex [38,39].

Physicochemical parameters such as the number and position of heavy atoms, topological polar surface area (TPSA), molar refractivity, water solubility (S), and lipophilicity parameter were assessed [40]. The pharmacokinetic parameters, including skin penetration (Log K_p_), brain penetration, P-glycoprotein substrate ability (P-gp substrate), and gastrointestinal absorption (GI), were computed for ibuprofen, IA and Co–IA using the Brain or Intestinal Estimated Permeation approach (BOILED-Egg model) [41,42] (Table 6).

**Figure 11:**
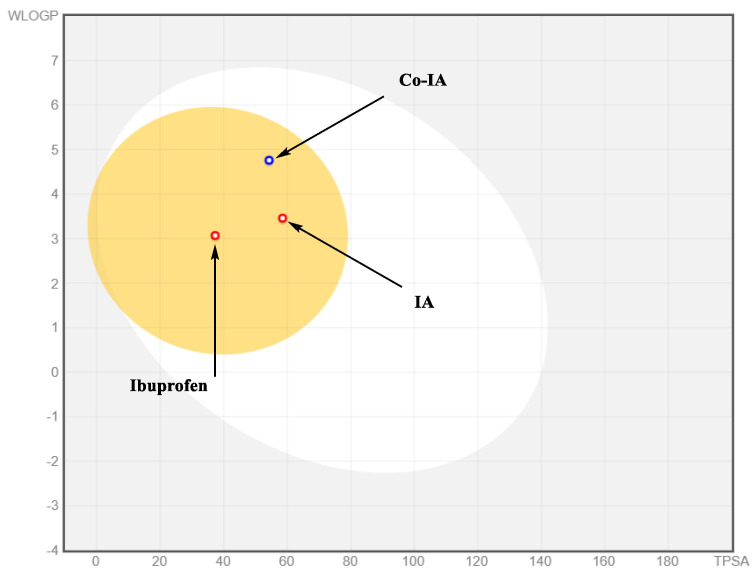
Ibuprofen, IA, and Co–IA BOILED-Egg model.

In the BOILED-Egg model, the yellow zone has a high chance of brain penetration, and the white zone has a substantial probability with GI absorption. The yolk and white areas do not have to be opposite each other. Additionally, the dots are colored blue when P-glycoprotein is predicted to be actively effluxed (PGP+) and red when P-glycoprotein is expected not to be a substrate. Ibuprofen, IA, and the Co–IA were demonstrated to be passively permeable through the BBB and to have high absorption through the GIT (Figure 11). 

The new compounds were evaluated using Veber’s rule-based approach (10 or fewer rotatable bonds and TPSA of 140 Å^2^ or less are associated with a high probability of good bioavailability) [43,44,45]. Table 6 indicates that compounds IA and Co–IA did not exhibit any violations and are therefore suitable therapeutic candidates for bioactivity research.

### 2.6. In Vitro: Anti-Inflammatory Action

#### 2.6.1. IA and Its Complexes: Cyclooxygenase Inhibition

**Figure 12:**
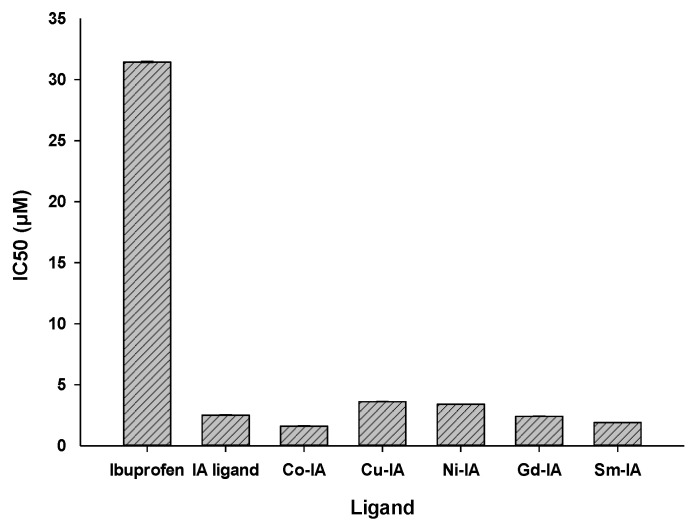
The IC_50_ of ibuprofen, IA, and complexes.

The compounds’ abilities were evaluated in vitro to inhibit ovine COX-1 and human recombinant COX-2 isozymes using ELISA [46]. Selectivity indexes for COX-2 (S.I. values = IC_50_ (COX-1)/IC_50_ (COX-2)) were also calculated and compared with the common drugs ibuprofen, diclofenac sodium and indomethacin. The data indicated that the IA blocked COX-1 (IC_50_ = 10.2 µM) and COX-2 (IC_50_ = 3.6 µM) more than ibuprofen (COX-1 (IC_50_ = 12.9 µM) and COX-2 (IC_50_ = 31.4 µM). IA had a higher S.I. (2.5) than ibuprofen (0.4) and was shown to become more efficient than ibuprofen at inhibiting COX-1/COX-2 (Table 7). The IC50 values of Cu, Ni, Co, Gd and Sm complexes were 3.4, 2.5, 2.4, and 1.9 µM, respectively, which indicates that they act as more potent COX-2 inhibitors than IA and ibuprofen (3.6, 31.4 µM), as shown in Table S6 and Figure 12.

#### 2.6.2. Western Blotting Assay

**Figure 13:**
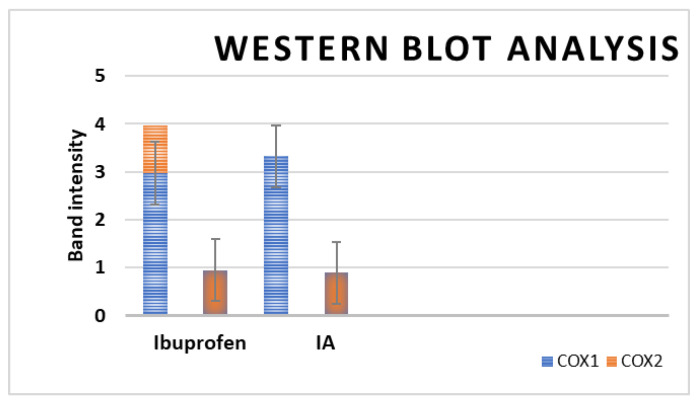
Histogram of ibuprofen and IA with *COX* enzymes.

**Figure 14:**
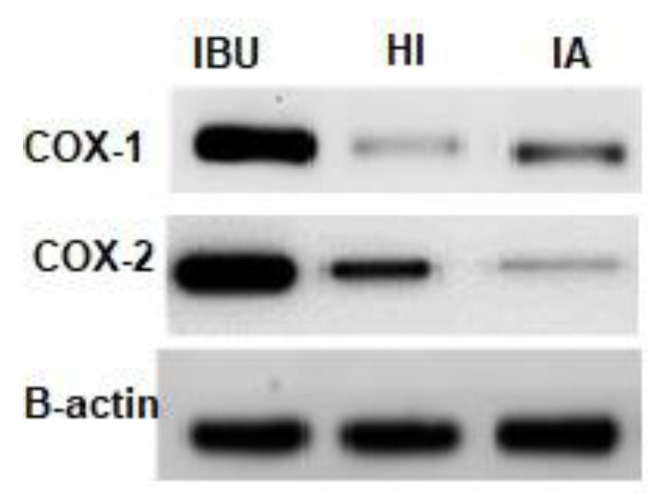
*COX* enzymes in a Western blot with IA.

Western blotting of COX-1/COX-2 verified IA’s anti-inflammatory activity [47,48]. The data showed that the IA had high potential as an inhibitor of COX-1 (IC_50_ = 0.946 µM), and COX-2 (IC_50_ = 0.894 µM (Figure 13 and Figure 14). The COX-2 selectivity index of IA was greater (1.058) than that of ibuprofen (0.895), which indicates that it is a more selective COX inhibitor than ibuprofen, as shown in Table 8.

#### 2.6.3. The MTT Test

The cytotoxicity of the IA was investigated during 24 h at various concentrations against fibroblast cell lines using the MTT test [49]. IA showed higher inhibitory action of COX-2 (IC50 = 3.43 µM) than ibuprofen (IC50 = 31.4 µM) (Table 9).

## 3. Materials and Methods

### 3.1. Materials

All chemicals and ibuprofen were obtained in their pure state (Sigma Aldrich, Schnelldorf, Germany), acetylacetone, nickel (II) chloride, copper (II) chloride, cobalt (II) chloride hexahydrate, gadolinium (III) nitrate hexahydrate, and samarium (III) nitrate hexahydrate. Highly polymerized fish-milt DNA (FM-DNA), Tris-HCl buffer, and pure HPLC-grade solvents were purchased from Sigma-Aldrich. All glassware and other items were dried after cleaning with distilled water before use.

### 3.2. Instrumentation

An electrical melting point apparatus was used to determine the melting points. A Xylem Analytics’ WTW digital conductivity meter (Weilheim, Germany) was employed on 10^−3^ M DMSO solutions to evaluate the electrical conductivity of the complexes at ambient temperature. The CHNS-932 (LECO) elemental analyzer was used to conduct microanalyses of C, H, and N. The complexes were digested with concentrated HNO_3_/H_2_O_2_ for metal analysis, and the remaining material was subsequently dissolved in distilled water. The metal ratio was determined by thermogravimetric analysis or EDTA titration. Using a Varian spectrometer and the DMSO-*d*_6_ solvent as a reference, the ^1^H NMR spectra were acquired, and the chemical shifts were quantified in ppm. Electronic spectra of the metal complexes were obtained using a quartz cuvette and a UV-1800 Shimadzu spectrophotometer (200–800 nm; 1 cm path length), and in a Nujol mull, following the method described by Lee et al. [50]. The 400–4000 cm^−1^ range was used to acquire the FTIR spectra of the ligand and its complexes using a Bruker Tensor 27 spectrophotometer (KBr disc). A Shimadzu Qp-2010 plus mass analyzer was used for mass spectrometry. The Gouy method was using mercury (II) tetrathiocyanatocobaltate (II) as the reference to evaluate the magnetic susceptibility using an MSB-MK1 balance at the ambient temperature. Thermal analysis (TGA, DTG, and DTA) was performed using a Shimadzu 60H thermal analyzer with a nitrogen flow of 20 mL/min and heating rate of 10 °C/min in the range of 40–800 °C. Chem Office (Version 16.0) was used to model the molecules of the novel compounds in order to use the MM2 calculation to carry out an energy optimization operation.

### 3.3. General Synthesis

We used a conventional approach that was employed frequently in the literature [5,51,52,53], with some variety in the time of reflux, to synthesize the ester ethyl 2-(4-isobutyl phenylopanoate (IE) and the hydrazine [2-(4-isobutylphenyl)propane hydrazide (HI)].

#### 3.3.1. Synthesis of IA

**Scheme 2:**
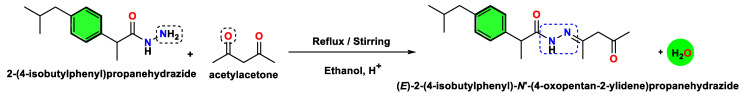
Synthesis of IA.

Ethanolic solutions of ibuprofen hydrazide (C_13_H_20_N_2_O) and acetylacetone were added dropwise, followed by heating of the mixture at reflux for three hours. The solution was allowed to evaporate completely, and only a trace of its original volume remained, which was then allowed to cool to room temperature. After filtration, washing with methanol, and drying, the canary-yellow precipitate was desiccated using anhydrous CaCl_2_ (Scheme 2).

#### 3.3.2. Synthesis of Metal Complexes

In a hot ethanolic solution, 0.1 mmol of CuCl_2_ (0.067 g), NiCl_2_ (0.0648 g), CoCl_2_·6H_2_O (0.1189 g), Sm(NO_3_)_3_·6H_2_O (0.222 g), or Gd (NO_3_)_3_·6H_2_O (0.225 g) was added dropwise to 0.2 mmol of ligand IA. After 2–3 h of vigorous stirring under reflux, the solution was allowed to cool to 25 °C. Co, Ni, and Cu complexes changed the final pH of the solution to 7–8, while Sm and Gd complexes changed it to 4–5.5. After heating, the solutions were allowed to evaporate slightly and then left to reach room temperature, and the complexes were formed. Filtration, washing with methanol, and drying with a desiccator were performed on the precipitate (solid crystals).

### 3.4. Anti-Inflammatory Action

#### 3.4.1. ELISA Test

In accordance with Cayman Chemical protocols, an ELISA test kit was used (Item No. 560131, Ann Arbor, MI, USA) [54,55]. The inhibition of bovine COX-1 and human recombinant COX-2 using hydrazide, IA, and certain complexes was shown.

#### 3.4.2. Western Blot Analysis

By using immunoblotting, we were able to successfully confirm the anti-inflammatory mechanism of the pattern of protein expression [47,48,56].

#### 3.4.3. The MTT Assay

The MTT Reagent was purchased from Biospes (Biospes, China, Cat n#BAR1005-1), and used according to the manufacturer’s instructions [49,57,58]. Twenty-four hours prior to the MTT experiment, breast cancer cell lines (MCF-7 Cell Line) were seeded at a concentration of 5 × 10^4^ cells per well in 96-well microplates to facilitate microplate adherence. Various agent concentrations were administered to the cultured cells. The cells were treated with 10 μL of MTT solution, and 100 μL of serum-free medium was added to every well. The plate was kept at 37 °C for 4 h in a CO_2_ incubator. Then, each well received 100 μL of formazan diluent buffer. The plate was incubated at 37 °C for the entire night after being wrapped in foil and given a 15-min orbital shake. An ELISA plate reader (Stat Fax 2200, Awareness Technologies, Palm City, FL, USA) was used to measure color absorbance in the OD range of 450–630 nm [59].

For every sample, the mean of the three readings was computed. Cell survival was calculated at the very end using the following equation:

(1) $$\%\;\mathrm{of}\;\mathrm{cell}\;\mathrm{viability}=\;\frac{Asample}{Acontrol}\times 100$$

A_control_ is the absorbance of the cell culture after treatment with formazan buffer and MTT without added any drug. A_sample_ is the equivalent absorption for samples that were treated concurrently with active substances. IC_50_ nonlinear regression and curve fitting were then used to determine the values [60].

## 4. Conclusions and Future Perspectives

IA, a new ibuprofen derivative, was prepared as a pure compound. The complexes formed by the reaction of the IA with Ni(II), Co(II), Cu(II), Sm(III), and Gd(III) were synthesized and characterized. The IA and the complexes often showed more bioactivity than the widely used drug ibuprofen. An octahedral structure is formed by Cu, Co, and Ni, whereas a capped square antiprismatic or tricapped trigonal prismatic structure is formed by Gd and Sm. The IA and its complexes had greater inhibitory ability towards COX-1/COX-2 than ibuprofen. The IA and complexes were docked with the COX-2 protein. The binding energy obtained for the in vitro investigations was compared and evaluated. The in silico tools produced promising results that were comparable to in vitro data, indicating that they can be employed to forecast the bioactivity of novel drugs. In vitro studies were performed on the novel compounds’ anti-inflammatory properties. The new compounds were found to have more potent action as inhibitors of inflammatory enzymes, suggesting that they could be introduced as a new drug following further experiments, including in vivo studies.

However, to fully establish the therapeutic viability and safety profile of IA and its complexes, future research is proposed to further enhance the bioactivity and reduce the potential side effects of IA. Therefore, the synthesis and evaluation of additional derivatives should be continued. Variations in the molecular structure might lead to compounds with even greater efficacy or specificity toward inflammatory pathways. Although in vitro and in silico analyses have shown promising results, in vivo studies are crucial to understanding the pharmacokinetics, pharmacodynamics, and toxicity profiles of these compounds in a living organism. Such studies will provide essential data on the efficacy, safety, and potential side effects of IA and its complexes. In addition to COX-1/COX-2 inhibition, exploring the effect of IA and its complexes on other biological pathways involved in inflammation could uncover additional mechanisms of action. This might include pathways like NF-κB, TNF-α, and various cytokines, which play significant roles in the inflammatory process. Further comparative studies with other NSAIDs and COX-2 inhibitors are needed to position IA and its complexes within the broader spectrum of anti-inflammatory drugs. This will help in identifying any unique advantages or disadvantages they may have over existing treatments.

## Figures and Tables

**Table 1:** Data analysis and measurement of conductivity for IA and its complexes.

Compound	Molecular Weight	Color	Melting Point (°C)	Ω * (µS)	CHNM%					
					C%	H%	N%	M% **		
					Calc.	Calc.	Calc.	A	B	Calc.
					Found	Found	Found			
IA (C_18_H_26_N_2_O_2_)	302.5	Canary-yellow	135	---	71.42 71.33	8.59 8.55	10.58 10.61	---	---	---
[Cu(IA)·(H_2_O)·Cl_2_]6H_2_O	562.97	Dirty green	>280	13	47.53 47.44	6.20 6.22	6.16 6.20	12.16	11.59	11.29
[Ni(IA)·(H_2_O)_2_·Cl]Cl·H_2_O	486.06	Violet	>280	100	49.98 49.88	6.99 6.88	6.48 6.51	12.28	12.08	12.08
[Co(IA)·(H_2_O)·Cl_2_]2H_2_O	468.29	Brick brown	>280	15	44.46 44.55	6.63 6.71	5.76 5.66	12.25	12.19	12.12
[Gd(IA)_2_·(NO_3_)_2_·(H_2_O)]NO_3_·2H_2_O	1002.25	Yellowish white	>280	10	47.23 47.22	5.89 5.92	9.44 9.50	---	15.75	15.69
[Sm(IA)_2_·(NO_3_)_2_]NO_3_·3H_2_O	995.15	Yellowish white	>280	100	48.59 48.61	5.83 5.91	9.71 9.80	---	15.09	15.15

* 10^−3^ M in DMSO, conductivity (ohm^−1^ cm^2^ mol^−1^). ** A is the metal percentage determined by EDTA titration and B is determined by thermal analysis.

**Table 2:** IR data for IA and the complexes (cm^−1^).

Compound	υ (NH, H_2_O, OH)	υ(C=O)	υ(C=N)	υ(NO_3_^−^)	υ (M-O)	υ(M-N)
IA(C_18_H_26_N_2_O_2_)	3720–3000 m, br	1725 s 1660 w	1611 s	---	---	---
[Cu(IA)Cl_2_·(H_2_O)]6H_2_O	3780–3000 m, br	1641w	1528 sh	---	636 w	540 w
[Ni(IA)·(H_2_O)_3_]Cl_2_·H_2_O	3760–3070 m, br	1608 w	1510 sh	---	622 w	521 w
[Co(IA)·(H_2_O)·Cl]Cl·H_2_O	3760–3070 m, br	1612 w	1508 sh	---	617 w	526 w
[Gd(IA)_2_·(H_2_O)·(NO_3_)]2H_2_O	3790–3070 m, br	1612 w	1528 sh	1384	651 w	531 w
[Sm(IA)_2_·(H_2_O)·NO_3_](NO_3_)_2_·2H_2_O	3760–3080 m, br	1617 w	1518 sh	1384	680 w	516 w

**m:** medium, **w:** weak, **s:** strong, **br**: broad, and **sh:** shoulder.

**Table 3:** TGA and DTG of the complexes of IA ligand.

Compound	Molecular Weight	Temp. Range °C	DTG Temp. °C	Mass Loss %		Process	Expected Products	Residue	M%
				Found	Calcd.			(Calcd.) Found	
[Cu(IA)·(H_2_O)·Cl_2_]6H_2_O	562.97	36–64	49	19.70	19.20	Dehydration	6H_2_O	CuO (14.15) 14.50	11.59
		180–310	304	15.14	15.16	Coordination sphere + ligand decomposition	2HCl+ H_2_O+ 0.15 IA		
		330–348	338	30.84	30.81	ligand decomposition	0.43 IA		
		395–460	423	20.75	20.56	Complete decomposition	0.37 IA		
[Ni(IA)·(H_2_O)_2_·Cl]Cl·H_2_O	486.06	38–58	49	3.65	3.71	Dehydration	H_2_O	NiO (15.37) 12.41	12.08
		289–347	341	50.46	50.01	Coordination sphere + ligand decomposition	2HCl + 2H_2_O + 0.51 IA		
		385–401	393	30.62	30.61	Complete decomposition	0.49 IA		
[Co(IA)·(H_2_O)·Cl_2_]2H_2_O	486.29	28–199	53	7.35	7.41	Dehydration	2H_2_O	CoO (15.40) 18.99	12.12
		263–321	271	46.62	46.56	Coordination sphere + ligand decomposition	2HCl+ H_2_O 0.49 IA		
		296–356	353	30.50	30.52	Complete decomposition	0.51 IA		
[Gd(IA)_2_·(NO_3_)_2_·(H_2_O)] NO_3_·2H_2_O	1002.25	24–35	38	4.21	3.59	Dehydration	2H_2_O	Gd_2_O_3_ (36.17) 33.25	15.75
		253–317	280	33.64	33.65	Coordination sphere + Ligand decomposition	H_2_O+ 2HNO_3_ 0.55 IA		
		398–434	420	15.82	15.92	Coordination sphere + ligand decomposition	HNO_3_ + 0.30 IA		
		481–607	497 548 590	9.97	9.98	Complete decomposition	0.17 IA		
[Sm(IA)_2_·(NO_3_)_2_]NO_3_·3H_2_O	995.25	69–134	98	5.45	5.34	Dehydration	2H_2_O	Sm_2_O_3_ (35.03) 30.01	15.09
		260–299	279	53.06	53.11	Coordination sphere + ligand decomposition	2HNO_3_ 0.80 IA		
		453–476	460	6.62	6.63	NO_3_ liberation + ligand decomposition	HNO_3_+ 0.16 IA		
		651–670	660	0.39	0.38	Complete decomposition	0.092 IA		

**Table 4:** The metal complex degradation steps’ chosen kinetic parameters.

Complex	Step	R^2^	Order (n)	Ts (K)	*E*a (J/mol)	*Z* (s^−1^)	Δ*S*^‡^ (J/K·mol)	Δ*H*^‡^ (kJ/mol)	Δ*G*^‡^ (kJ/mol)
[Cu(IA)(H_2_O)Cl_2_]6H_2_O	Complete ligand decomposition	0.97	2.00	696	231.38	2.87 × 10^17^	+82.26	−5.555	−62.81
[Ni(IA)(H_2_O)_2_Cl]Cl·H_2_O	Coordination sphere+ ligand decomposition	0.99	2.00	614	32.69	212.24	−206.41	−5.072	121.05
[Co(IA)(H_2_O)Cl_2_]2H_2_O	Complete ligand decomposition	0.99	2.00	626	45.83	1.37 × 10^4^	−172.10	−5.0159	102.58
[Gd(IA)_2_(NO_3_)_2_(H_2_O)]NO_3_·2H_2_O	NO_3_ liberation and ligand decomposition	0.98	2.00	693	172.49	1.02 × 10^13^	−2.88	−5.589	−3.59
[Sm(IA)_2_(NO_3_)_2_]NO_3_·3H_2_O	NO_3_ liberation and ligand decomposition	0.99	2.00	552	115.17	1.19 × 10^10^	−57.16	−4.474	27.08

**Table 5:** The interaction parameters of ibuprofen and their derivatives IA with *COX-2*.

Compound	Ligand Sites	Receptor Sites	Type of Interaction	Bond Distance (Å)	Binding Energy (kcal/mol)	(S) (kcal/mol)
Ibuprofen	O(14)	THR 212	H-donor	2.85	−2.9	−5.33
IA	N(14)	TYR 385	H- donor	3.05	−0.7	−6.48
Co–IA	C(1)	HIS214	H- acceptor	3.19	−5.2	−6.65
	Ph	HIS207	H-pi	3.78	−0.6	
	Ph	HIS388	H-pi	3.70	0	
Cu–IA	Cl	SER 451	H- acceptor	3.47	−0.9	−7.45
	Ph	TYR385	H-pi	4.6	−0.6	
Ni–IA	O(21)	PHE 210	H- donor	2.73	−2.2	−8.16
	O(22)	ASN382	H- donor	3.19	−1.3	
	Ph	HIS 386	H-pi	4.69	−0.6	
Gd–IA	Ph	HIS 386	H-pi	4.42	−0.9	−7.41
Sm–IA	Ph	HIS 386	H-pi	4.33	−1	−9.14
	Ph	HIS 207	H-pi	4.86	−0.7	

**Table 6:** Predicted physicochemical variables for ibuprofen, IA, and Co–IA.

Compound	Ibuprofen	IA	Co–IA
Heavy atoms	15	22	26
Rotatable bonds	4	8	4
H-bond donor	1	1	1
H-bond acceptor	2	3	4
Fraction Csp3	0.46	0.50	0.39
LogS	−3.44	−5.27	−6.13
XLogP3	3.50	3.35	6.82
Molar refractivity	62.18	91.33	111.07
TPSA (Å^2^)	37.30	58.53	43.29
Log Kp (skin permeation) (cm/s)	−5.07	−5.77	−4.19
GI absorption	High	High	High
P-gp substrate	No	No	Yes

**Table 7:** Inhibition of cyclooxygenase by IA and standard drugs.

Compound	IC_50_ ^(a)^ (µM)		
	COX-1	COX-2	COX-2 ^(b)^ S.I.
Ibuprofen	12.9	31.4	0.4
Indomethacin	0.4	0.1	5.0
Diclofenac sodium	3.8	0.8	4.5
IA	10.2	3.6	2.8

^(a)^ IC_50_ The value represents the compound concentration required to inhibit COX-1 or COX-2 by 50%, which is the average of three determinations with a 10% deviation from the mean value. ^(b)^ Selectivity index (COX-1 IC50/COX-2 IC50).

**Table 8:** IA and ibuprofen inhibit cyclooxygenase through Western blotting.

Compound	IC_50_ (µM)		
	COX-1	COX-2	COX-2 S.I.
Ibuprofen	2.975	3.325	0.895
IA	0.946	0.894	1.058

**Table 9:** The IC_50_ (µM) of IA against fibroblast cells.

Compound	Fibroblast Cells
Ibuprofen	31.4
IA	3.43

## Data Availability

Data contained within the article.

## Supplementary Materials

The following supporting information can be downloaded at: https://www.mdpi.com/article/10.3390/ijms25063558/s1.
