# Supplementary material for: Design, Synthesis, Anti-Inflammatory Activity, DFT Modeling and Docking Study of New Ibuprofen Derivatives
Source: Int J Mol Sci. 2024 Mar 21;25(6):3558. doi: 10.3390/ijms25063558 (PMC10971055; doi:10.3390/ijms25063558)
Supplement: Supplementary file 1 [file ijms-25-03558-s001.zip › ijms-2862801-supplementary.pdf]

Table S1: The ^{1}H NMR data of the HI and IA.

<table>
<tr><th>Comp.</th><th colspan="2">δ (ppm)</th></tr>
<tr><td rowspan="3">HI</td><td colspan="2">9.13(1H, br, NH), 7.21-7.04 (4H, m, ArH), 4.18(2H, s, NH$_2$), 3.45(2H, s, CH$_2$), 2.39 (2H, s, CH$_2$), 1.80(1H, s, CH), 1.29 (3H, m, CH$_3$), 0.84-0.82(6H, m, (CH$_3$)$_2$).</td></tr>
<tr><td>Δ (ppm) map (existing)</td><td>δ (ppm) map (analyzed)</td></tr>
<tr><td>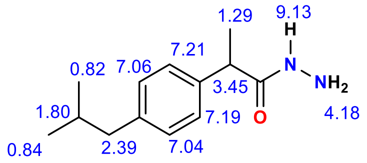
</td><td>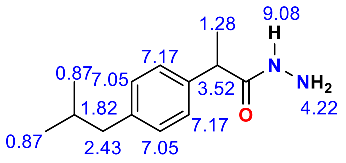
</td></tr>
<tr><td rowspan="4">IA</td><td colspan="2">δ (ppm)</td></tr>
<tr><td colspan="2">9.95(1H, br, NH), 7.24-7.05 (4H, m, ArH), 3.59(2H, s, CH$_2$), 3.31(2H, s, CH$_2$), 2.44(2H, s, CH$_2$), 2.44(2H, s, CH$_2$), 2.40 (3H, m, CH$_3$), 2.39 (3H, m, CH$_3$), 2.12(H, s, CH), 0.85-0.83(6H, m, (CH$_3$)$_2$).</td></tr>
<tr><td>Δ (ppm) map (existing)</td><td>δ (ppm) map (analyzed)</td></tr>
<tr><td>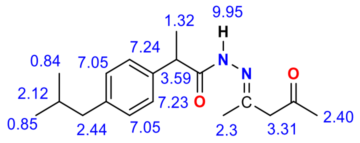
</td><td>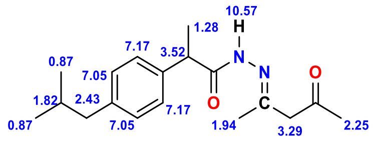
</td></tr>
</table>

*ppm: part per million, **δ** =shift value of ^{1}HNMR

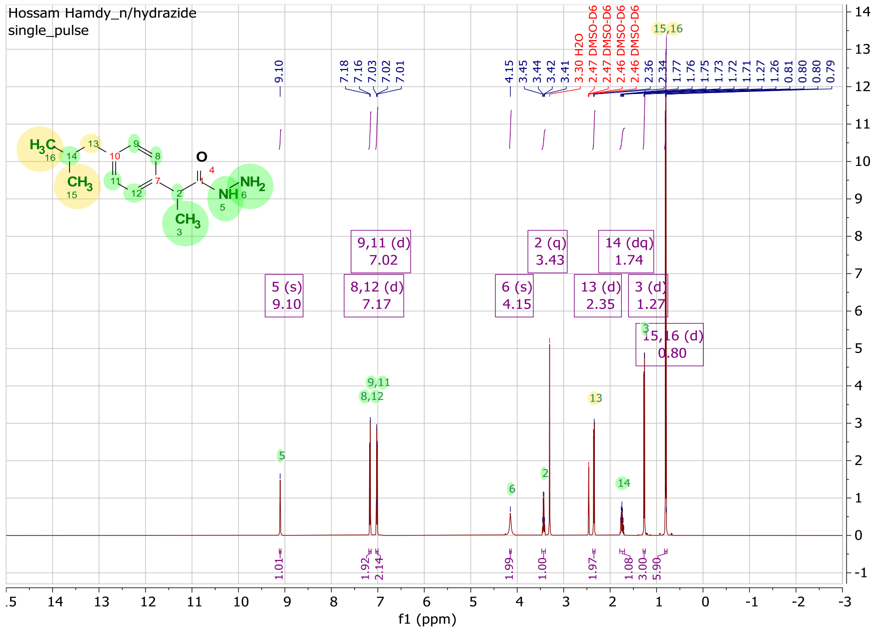

Figure S1: ^{1}HNMR spectrum of Hydrazide Ibuprofen **(HI)**.

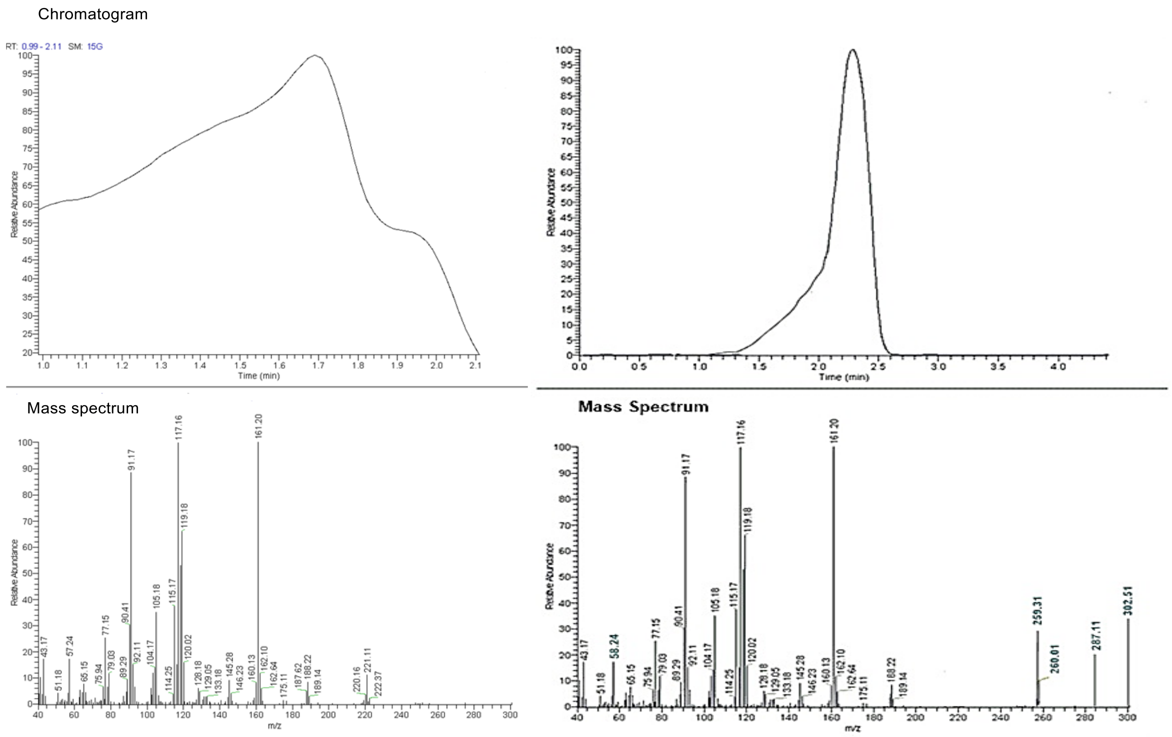

Figure S2: Chromatogram and mass spectrum of **HI** (on Left) and **IA** (on Right).

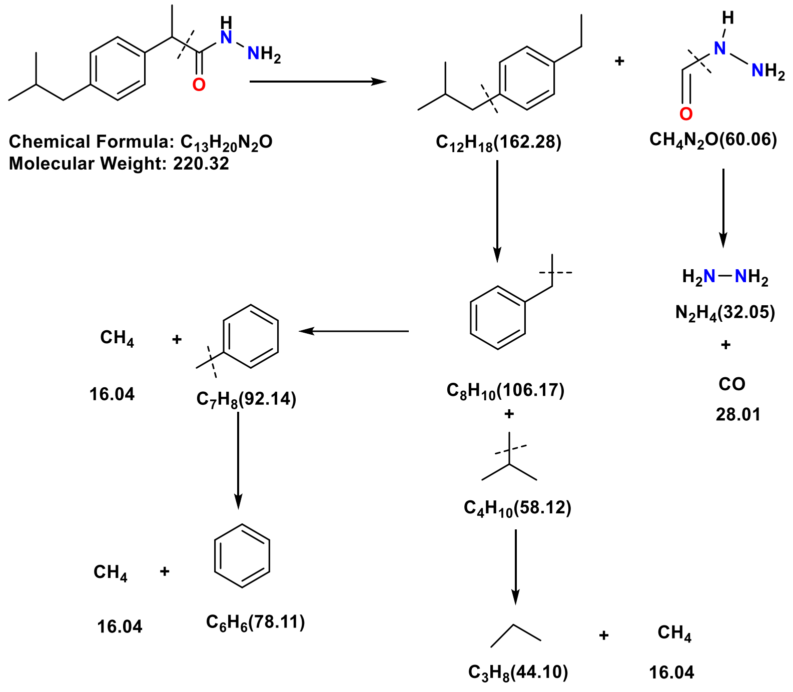

Scheme S1: The chromatogram of pathway fragmentation of Hydrazide Ibuprofen (**HI**).

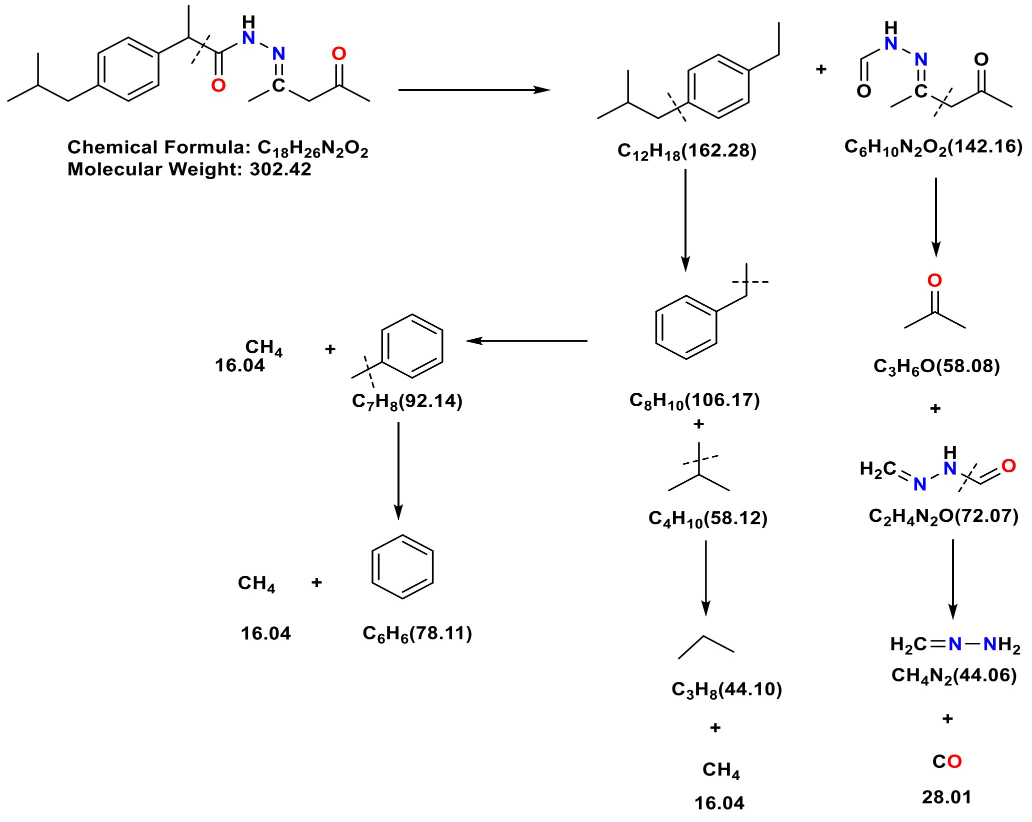

Scheme S2: The chromatogram of pathway fragmentation of **IA** ligand**.**

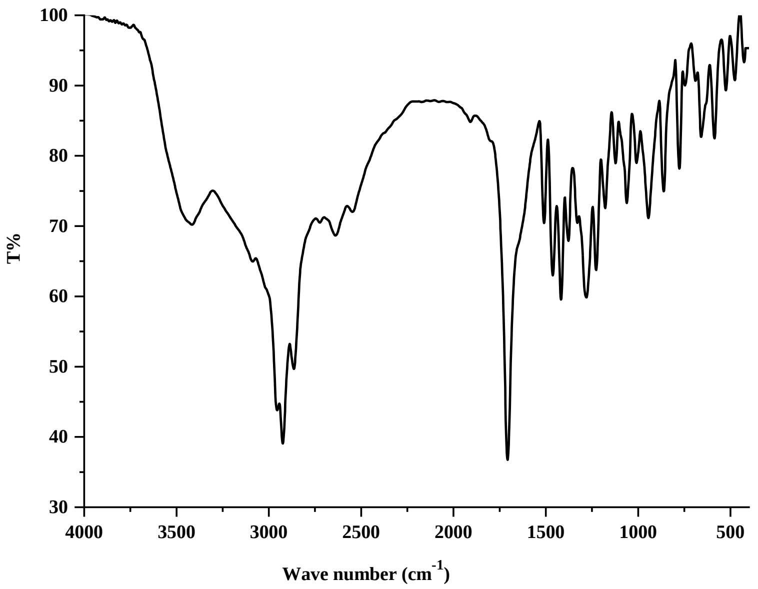

Figure S3: FTIR spectrum of the parent drug (**ibuprofen**).

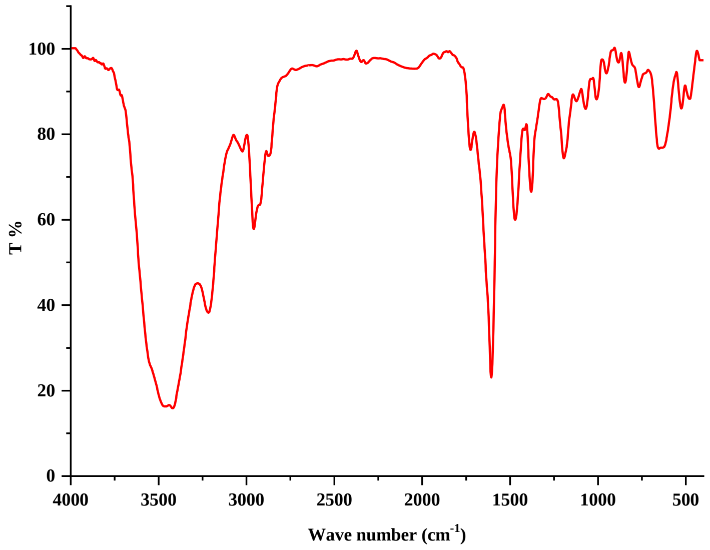

Figure S4: FTIR spectrum of the ligand **IA.**

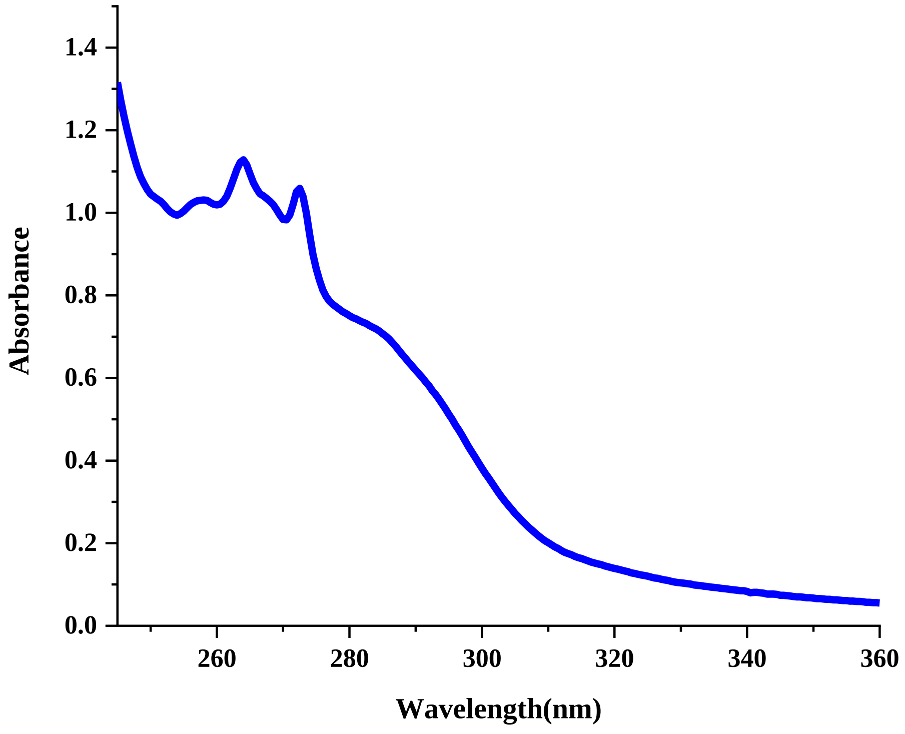

Figure S5: UV-Vis. spectrum of the ligand **IA**.

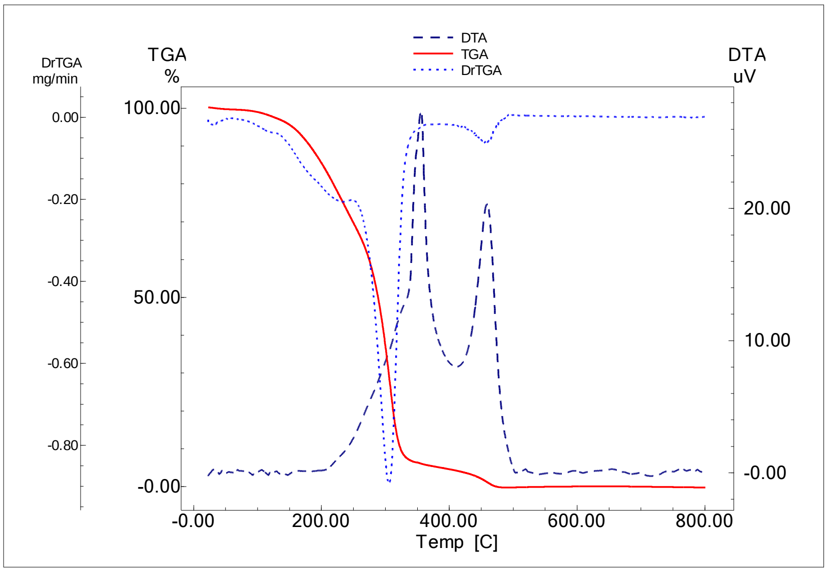

Figure S6: Thermal decomposition of Schiff base IA.

Table S2: TG/DTG and DTA data of IA.

Compound	***Temp. range*** **°C**	***DTA peak temp.*** **°C**	***Peak type***	***ΔH (KJ/g)***	***Process***
**IA**	111.7-136	130	Endo	0.017	Ligand decomposition
	346-367	355	Exo	-0.401	Ligand decomposition
	436-480	460	Exo	-0.0147	Final decomposition

Compound	***Temp. range °C***	***DTG Temp. °C***	***Mass loss %***		***Process***	***expected products***
			Found	***Calculated***		
**IA**	261-321	306	96.61	95.04	Ligand decomposition	0.95 IA
	443-473	458	3.68	4.96	Final decomposition	0.50 IA

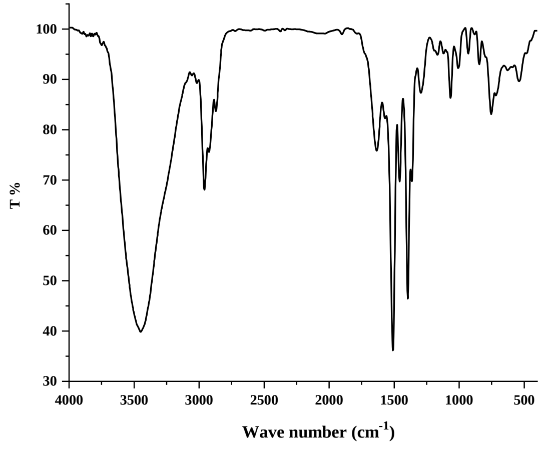

Figure S7: FTIR spectrum of [Cu(IA)Cl_2.H_2O]6H_2O.

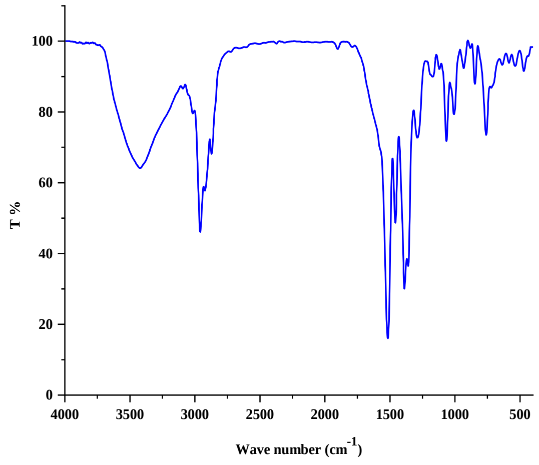

Figure S8: FTIR spectrum of [Ni(IA)$(H_2O)_3$]Cl_2.H_2O.

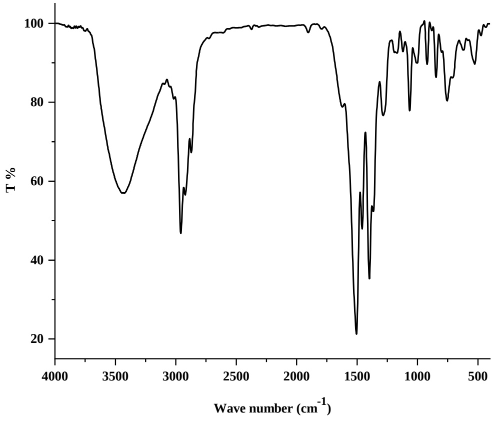

Figure S9: FTIR spectrum of [Co(IA)H_2OCl]Cl.H_2O.

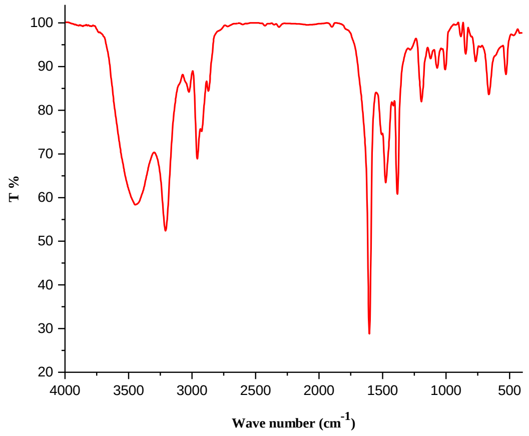

Figure S10: FTIR spectrum of [Gd$(IA)_2$(H_2O)(NO_3)]2H_2O.

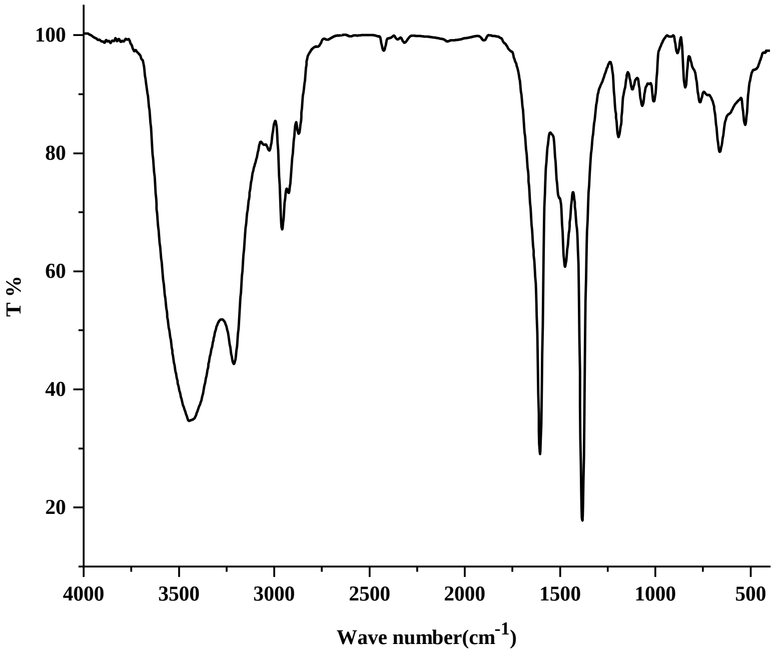

Figure S11: FTIR spectrum of [Sm(IA)$_2$H$_2$O NO$_3$](NO$_3$)$_2$.2H$_2$O.

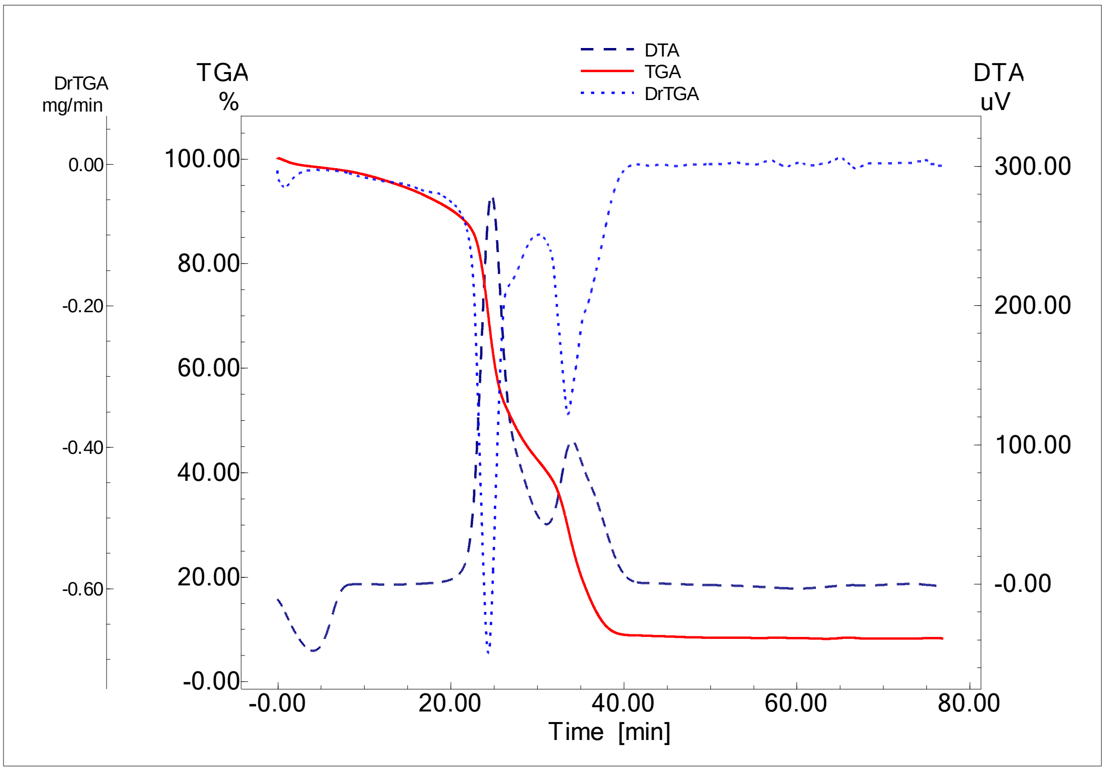

Figure S12: TGA/TG and DTA curves of [Ni(IA)(H$_2$O)$_2$ Cl]Cl.H$_2$O complex.

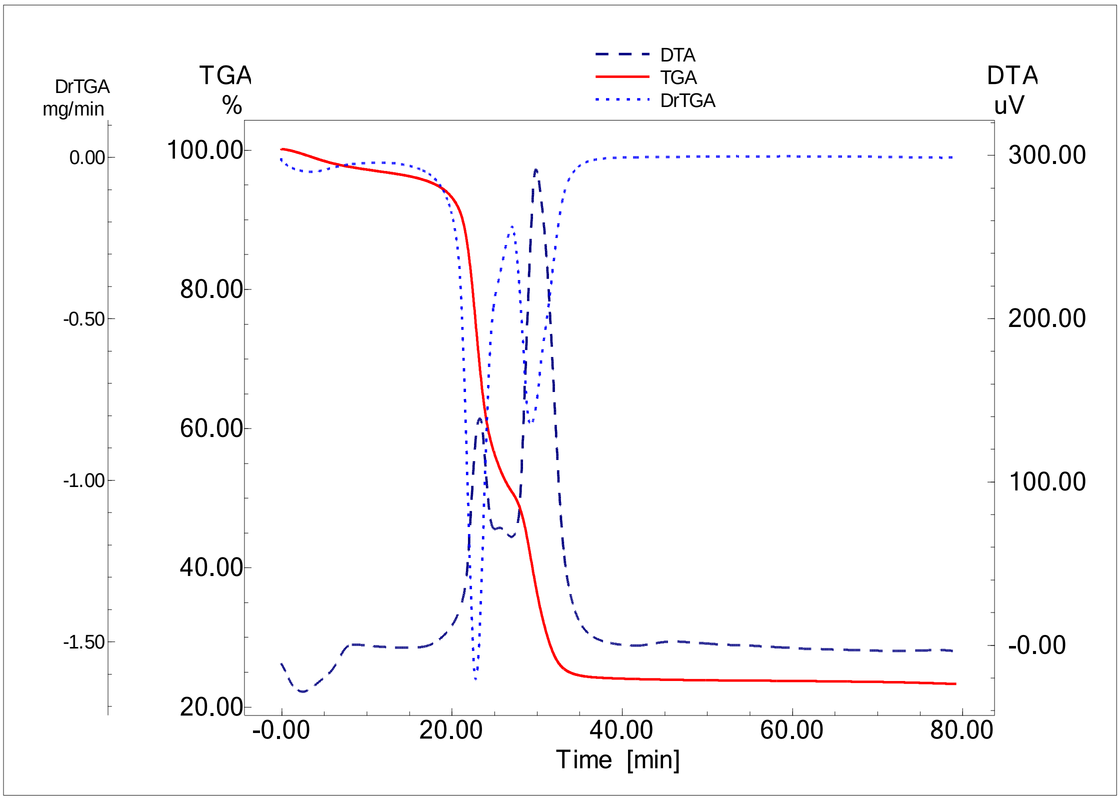

Figure S13: TGA/TG and DTA curves of [Co(IA).H_2O.Cl_2] $2H_2O$ complex.

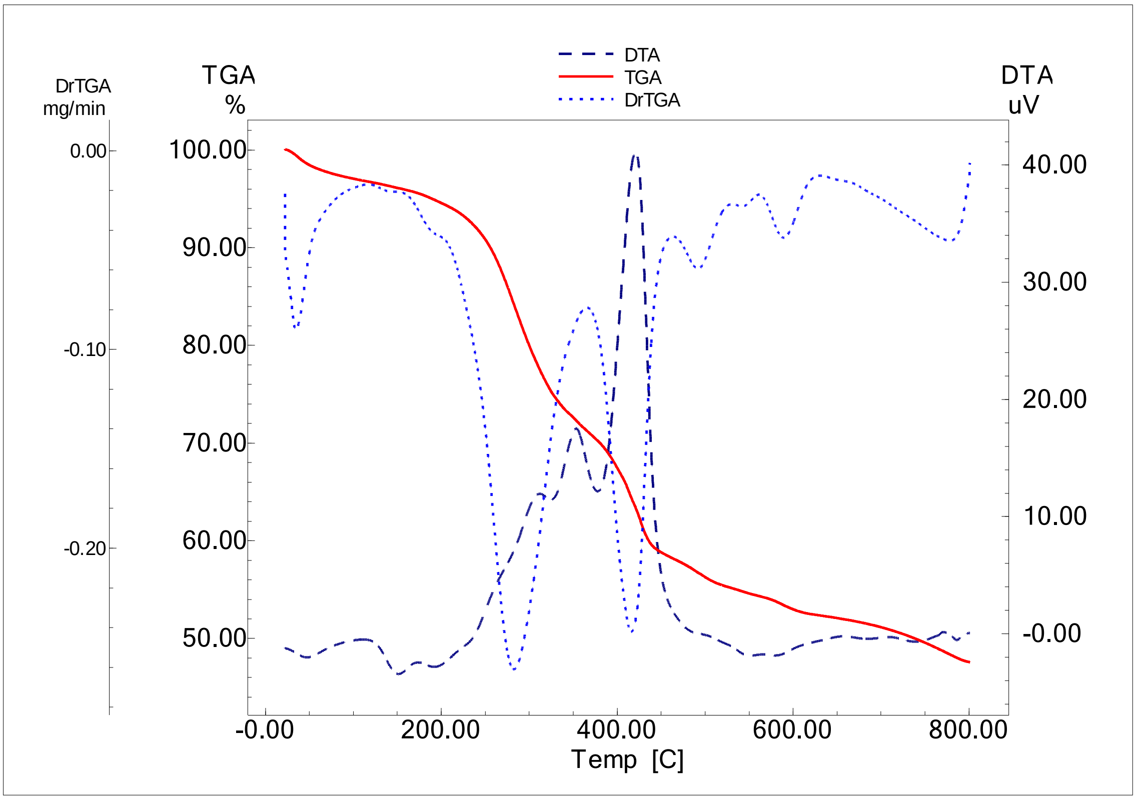

Figure S14: TGA/TG and DTA curves of [Gd(IA)$_2$(NO_3)$_2$ (H_2O)]NO_3.$2H_2O$ complex.

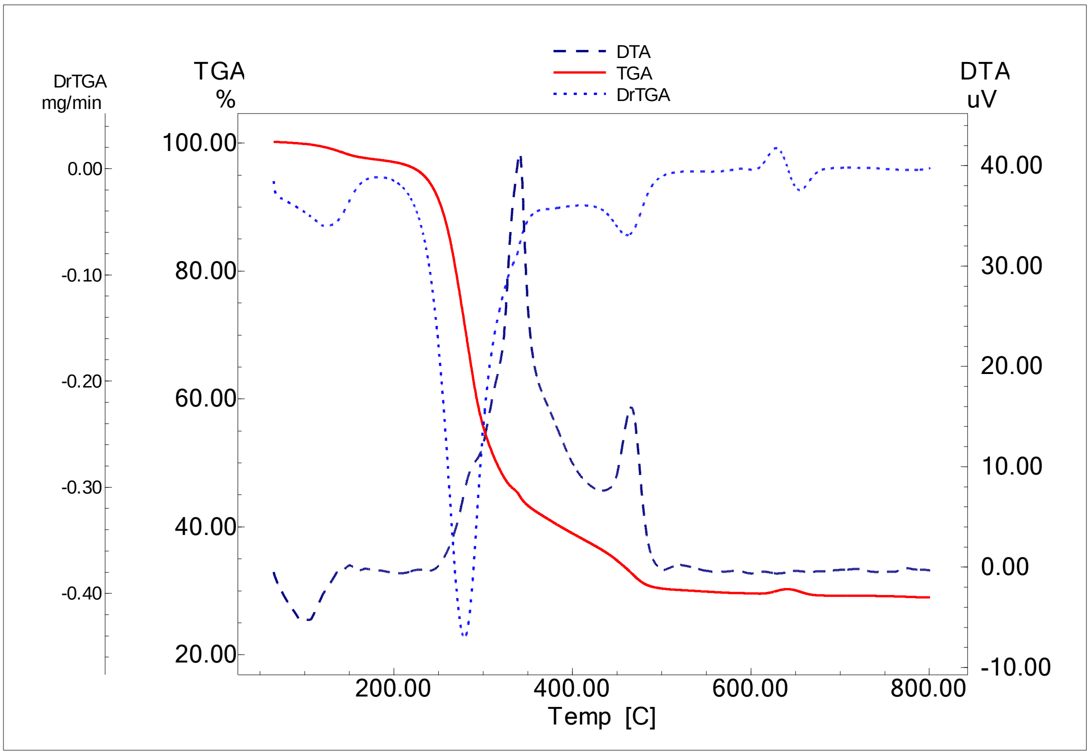

Figure S15: TGA/TG and DTA curves of $[Sm(IA)_2(NO_3)_2]NO_3.3H_2O$ complex.

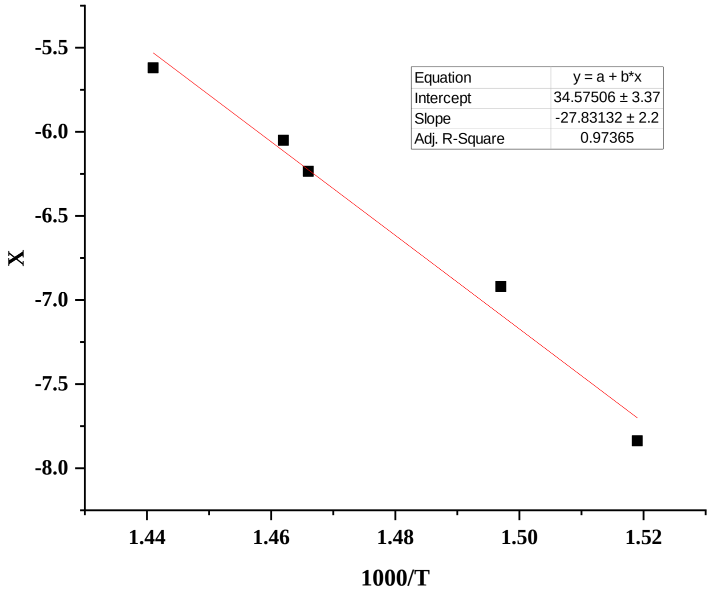

Figure S16: Fit-linear curve of liberation of coordinate water step of **Cu-L2** complex.

Table S3: DTA of Complexes derived from IA Schiff base.

Compound	***Temp. range °C***	***DTA peak temp °C***	***Peak type***	***ΔH (KJ/g)***	***Process***
$[Cu(IA)(H_2O)\ Cl_2]6H_2O$	28-78	56	Endo	0.550	Dehydration
	284-328	308	Exo	-0.851	Coordination sphere+ Partial Ligand decomposition
	341-356	350	Exo	-0.204	Ligand decomposition
	389-437	423,434	Exo	-3.47	Final decomposition
$[Ni(IA)(H_2O)_2\ Cl]Cl.H_2O$	46-164	86	Endo	2.29	Dehydration
	289-362	352	Exo	-11.11	Coordination sphere+ Ligand decomposition
	387-437	400,403	Exo	-3.68	Final decomposition
$[Co(IA)\ (H_2O)\ Cl_2]\ 2H_2O$	25-198	46	Endo	0.518	Dehydration
	251-280	273	Exo	-1.26	Coordination sphere+ Ligand decomposition
	319-360	351	Exo	-3.36	Final decomposition
$[Gd(IA)_2(NO_3)_2\ (H_2O)]\ NO_3.2H_2O$	133-161	48	Endo	0.018	Dehydration
	340-367	352	Exo	-0.382	Coordination sphere+ Ligand decomposition
	397-445	424	Exo	0.710	Ligand decomposition
	487-510	499	Exo	-0.069	Final decomposition
$[Sm(IA)_2(NO_3)_2]NO_3.3H_2O$	56-151	97	Endo	0.050	Dehydration
	330-350	340	Exo	-2.71	Ligand decomposition
	452-482	466	Exo	-0.234	Ligand decomposition
	510-550	512	Exo	-0.109	Final decomposition

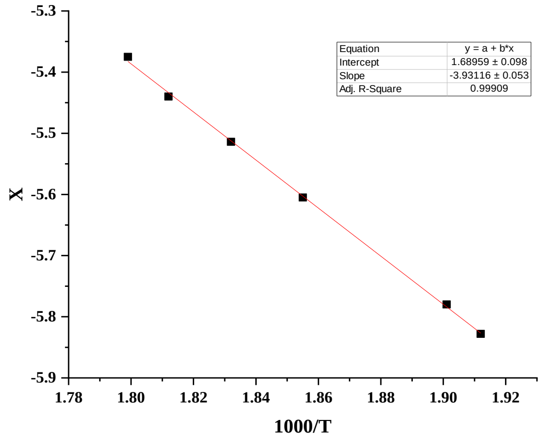

Figure S17: Fit-linear curve of liberation of coordinate water step of **Ni-IA** complex.

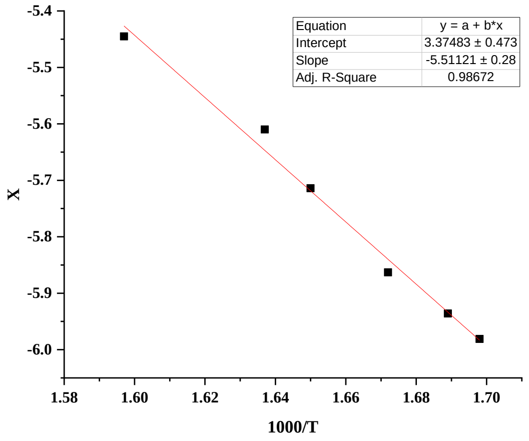

Figure S18: Fit-linear curve of liberation of coordinate water step of **Co-IA** complex.

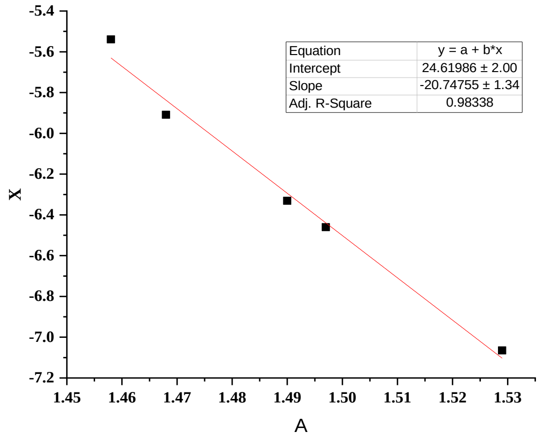

Figure S19: Fit-linear curve of liberation of coordinate water step of **Gd-IA** complex.

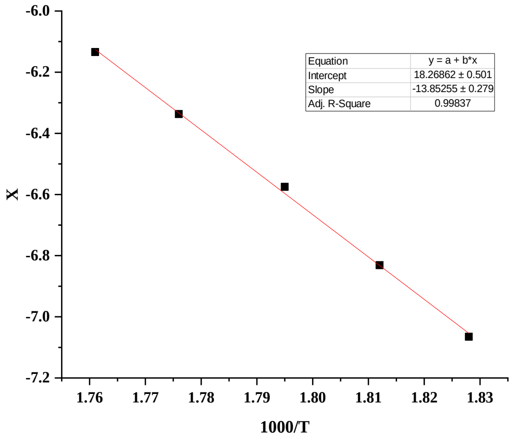

Figure S20: Fit-linear curve of liberation of coordinate water step of **Sm-IA** complex.

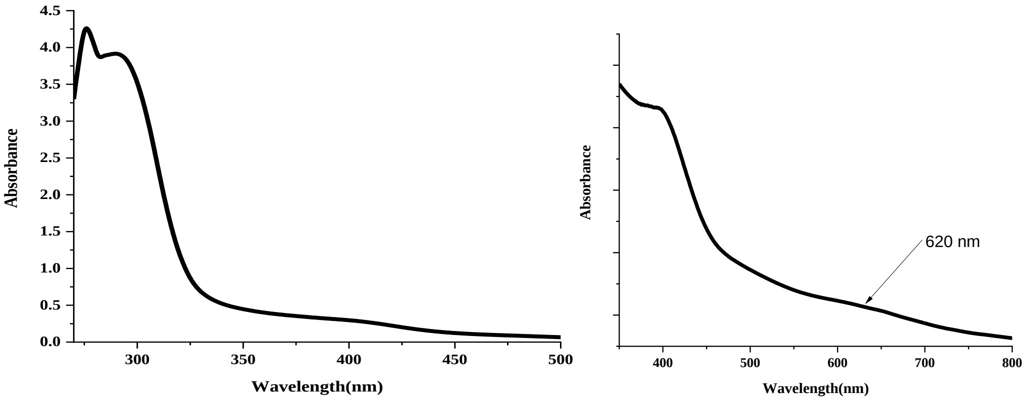

Figure S21: UV-Vis. spectrum of Ni-IA.

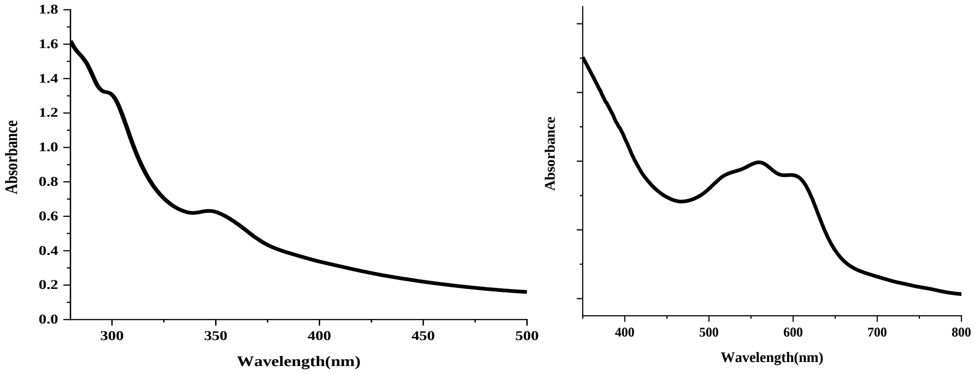

Figure S22: UV-Vis. spectrum of Co-IA.

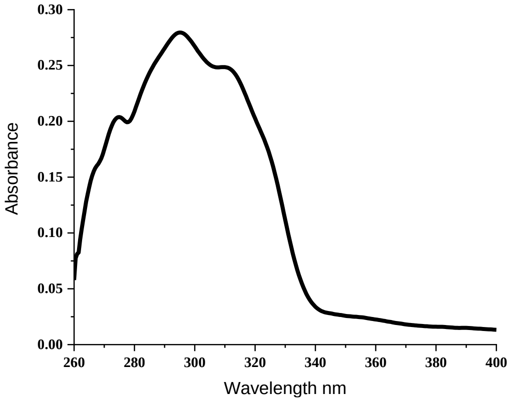

Figure S23: UV-Vis. spectrum of Gd-IA

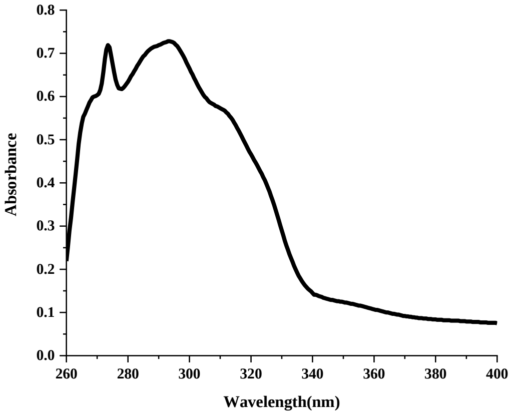

Figure S24: UV-Vis. spectrum of Sm-IA

Table S4: The magnetic properties and electronic spectra of IA ligand and its complexes

Comp.	***Peak***		***ε**** ***($M^{-1}cm^{-1}$)*** x10^4	***Assignment***	***10Dq***		***μeff*** ***(B.M)***	***Postulated Structure***
	nm	***cm^{-1}***			***cm^{-1}***	***kJ/mol***		
IA	257	38910	10.34	$\pi \rightarrow \pi^*$	---	---	---	---
	264	37878	11.27					
	272	36764	10.59	$n \rightarrow \pi^*$				
Cu-IA	267	37453	41.28	$\pi \rightarrow \pi^*$	25974	315	2.07	Distorted octahedral
	280	35714	38.58					
	351	28490	4.30	$n \rightarrow \pi^*$				
	385	25974	2.92					
	580	17241	42.53	$t_{2g} \rightarrow e_g$				
Ni-IA	276	36231	39.16	$n \rightarrow \pi^*$	24875	302	2.46	Distorted octahedral
	290	34482	4.33					
	402	26385	2.92	$^3A_{2g} \rightarrow {}^3T_{1g}(P)$				
	620	24875	13.18	$^3A_{2g} \rightarrow {}^1E_g$				
Co-IA	298	33557	6.32	$n \rightarrow \pi^*$	16583	201	4.86	Distorted octahedral
	347	28818	2.79	$^4T1g(F) \rightarrow {}^4T2g(P)$				
	520	19230	2.98	$^4T1g(F) \rightarrow {}^4A2g(F)$				
	558	17621	2.78					
	603	16583	2.03					
Gd-IA	275	36363	2.80	$\pi \rightarrow \pi^*$	---	---	8.89	Capped square antiprismatic / Tricapped trigonal prismatic
	295	33898	2.49	$n \rightarrow \pi^*$				
	311	32154	7.14	CT				
Sm-IA	273	36630	7.29	$\pi \rightarrow \pi^*$	---	---	2.29	Bicapped trigonal prismatic / Square antiprismatic
	293	34129	5.74	$n \rightarrow \pi^*$				
	310	32154	10.34	CT				

* ε= Absorptivity, 10^{-5} M in DMSO, $M^{-1}cm^{-1}$

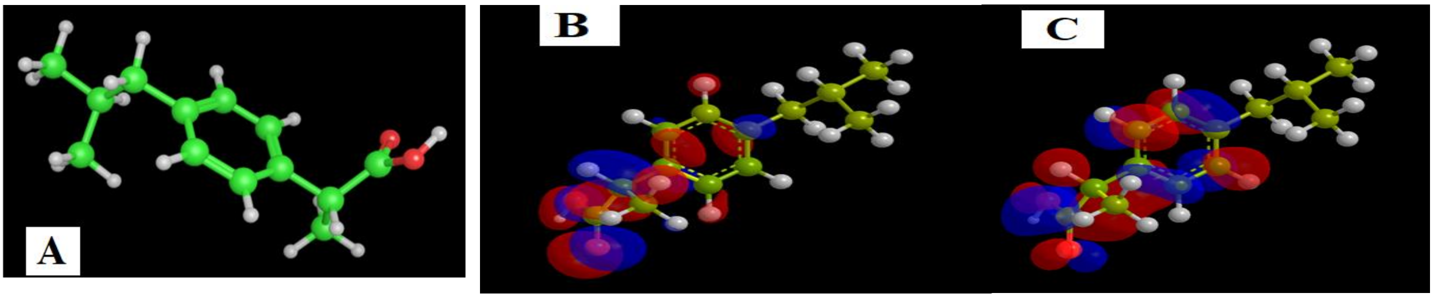

Figure S25: The DFT simulation for the Ibuprofen drug. **[A]** 3D view, **[B]** HOMO and **[C]** LUMO.

Table S5: The DFT simulation data and ligand propertied of IA.

Compound	***Ibuprofen***	**IA**
Formula	$C_{13}H_{18}O_2$	$C_{18}H_{26}N_2O_2$
Atoms	33	48
Orbitals	78	114
Electrons	82	120
SCF energy (E_h)	-93.85	-136.49
Dipole moment (D)	1.94	2.71
E_{LUMO} (E_h)	0.007	0.009
E_{HOMO} (E_h)	-0.349	-0.305
$\Delta E_{LUMO\text{-}HOMO}$ (E_h)	0.356 (128 nm)	0.314 (145 nm)
Ionization energy (E_h)	0.348	0.305
electron affinity (E_h)	-0.007	-0.009
Absolute electronegativity (E_h)	0.1705	0.148
Absolute hardness (E_h)	0.211	0.158
Absolute softness (E_h^{-1})	5.634	6.369
Global softness (E_h^{-1})	2.370	3.173
Global electrophilicity (ω)	0.045	0.069
Chemical potential (E_h)	-0.138	-0.148
Additional electronegativity (E_h)	0.654	0.937
Toxic.	no	no
Rsynth (%)	66.67	68.2
Weight (g/mol)	206.28	302.436
	37.30	58.53
TPSA	HD 1, HA 2	HD 1, HA 3
Log p	3.07	3.46
Log S	-3.64	-4.58

* **SCF energy**: Self-consistent field, **I**: Ionization potential, **Rsynth (%)**: Resynthesized %, **TPSA**: Topological polar surface area, **HD**: Hydrogen donor, **HA**: Hydrogen acceptor, **Logp**: Lipophilicity parameter and **Log S**: Water solubility parameter.

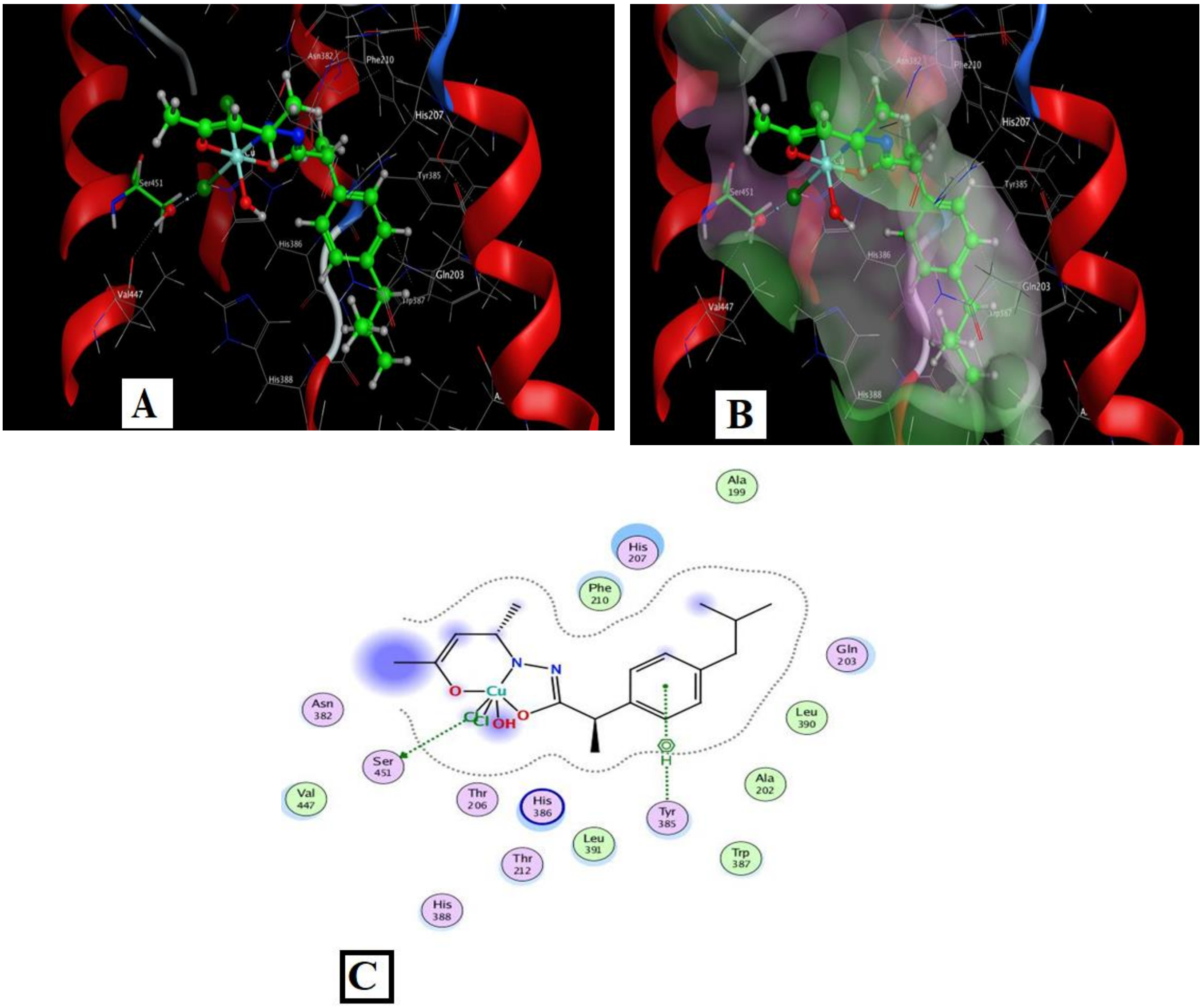

Figure S26: Docking model of the interaction of **Cu-IA** with ***Cox2*** [PDB code: 5IKT] bonding sites: (A) 3D interaction diagram (B) The surface properties [Hydrophilic sites (violet color), neutral sites (white color) and lipophilic sites (green color)]. (C) 2D interaction diagram

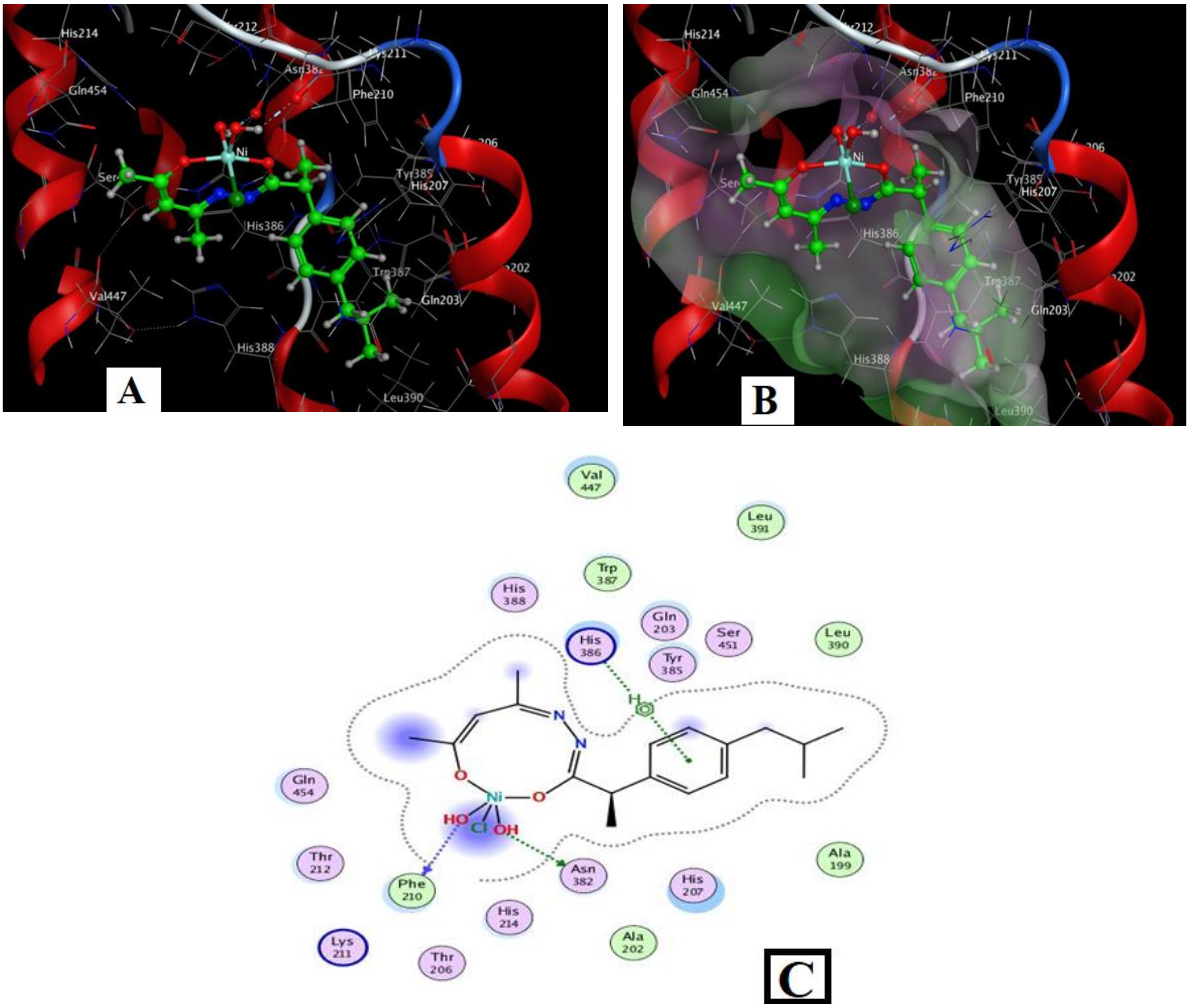

Figure S27: Docking model of the interaction of **Ni-IA** with ***Cox2*** [PDB code: 5IKT] bonding sites:

(B) 3D interaction diagram (B) The surface properties [Hydrophilic sites (violet color), neutral sites (white color) and lipophilic sites (green color)]. (C) 2D interaction diagram

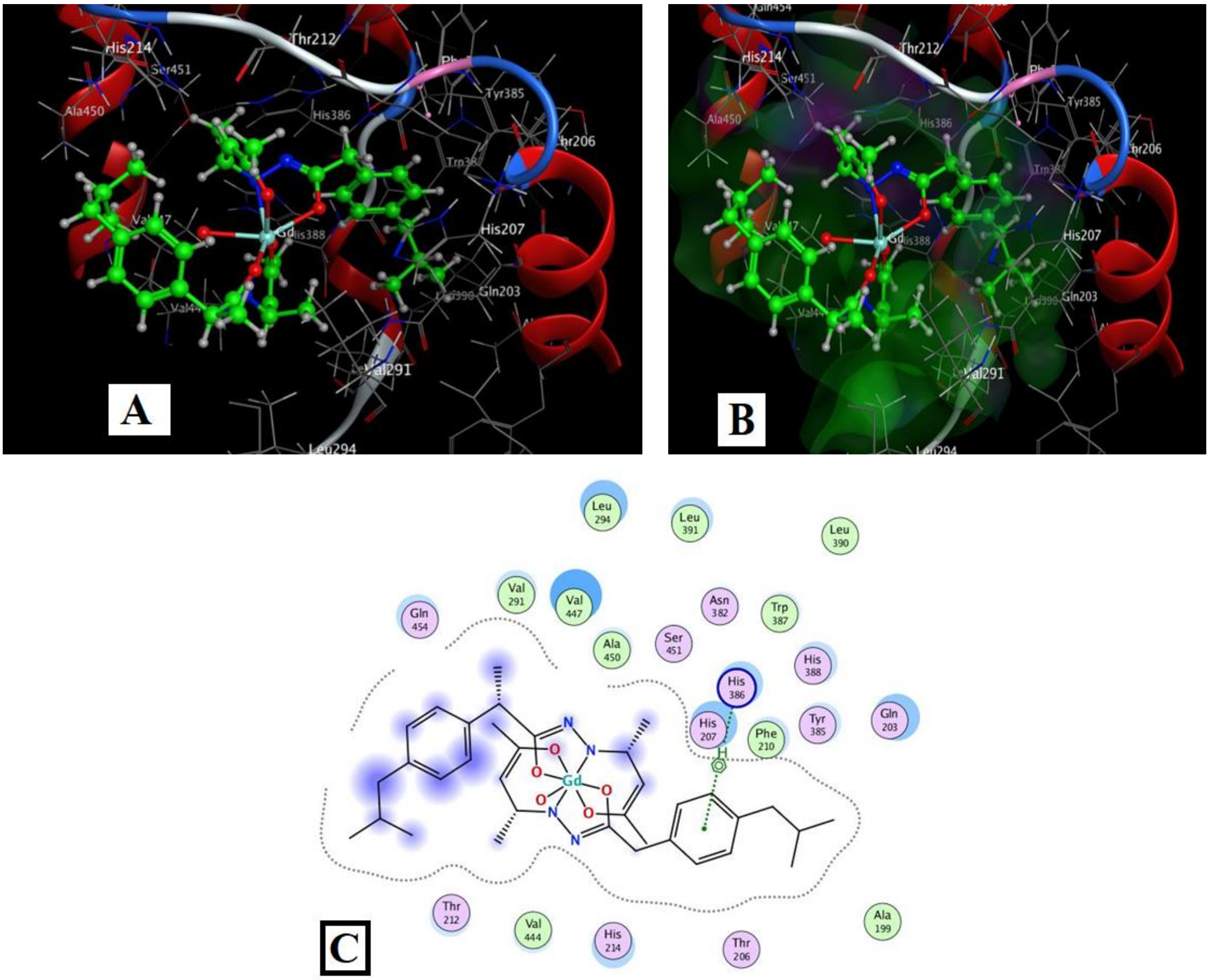

Figure S28: Docking model of the interaction of Gd-IA with ***Cox2*** [PDB code: 5IKT] bonding sites:

(C) 3D interaction diagram (B) The surface properties [Hydrophilic sites (violet color), neutral sites (white color) and lipophilic sites (green color)]. (C) 2D interaction diagram

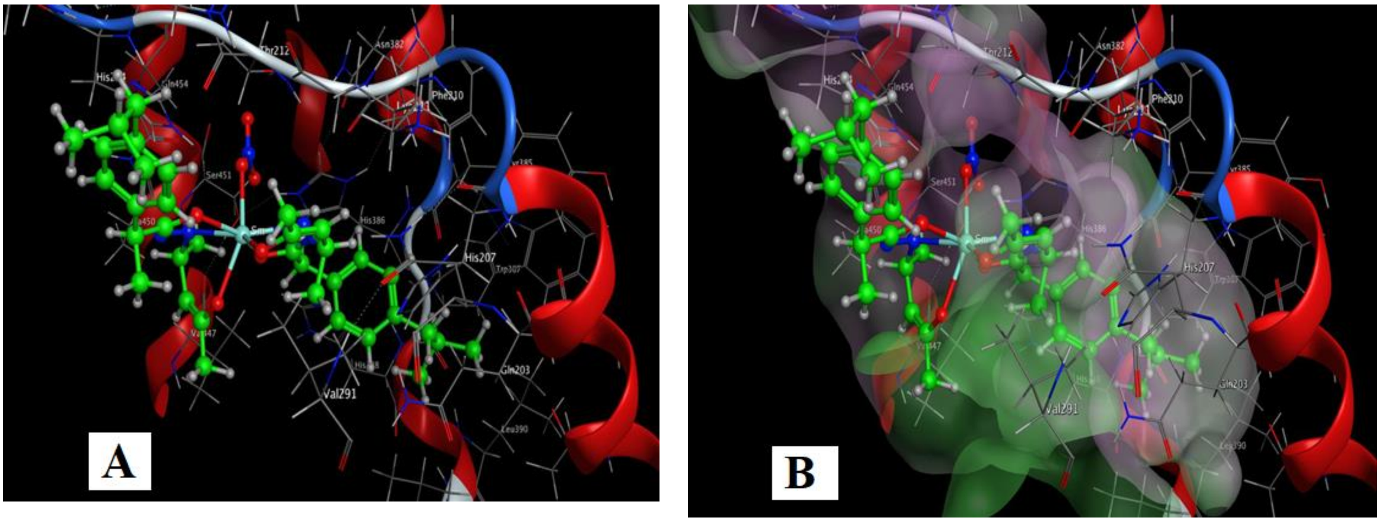

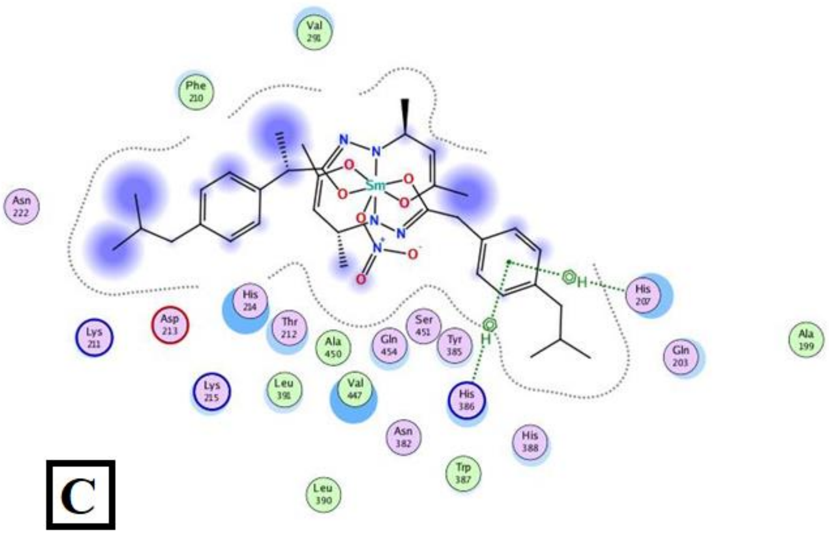

Figure S29: Docking model of the interaction of **Sm-IA** with ***Cox2*** [PDB code: 5IKT] bonding sites: (D) 3D interaction diagram (B) The surface properties [Hydrophilic sites (violet color), neutral sites (white color) and lipophilic sites (green color)]. (C) 2D interaction diagram

Table S6: In vitro COX-1 and COX-2 inhibition of different complexes of IA Schiff base.

**Compound**	**IC_{50} COX-2 (μM) [a]**
**Ibuprofen**	31.4
**Indomethacin**	0.1
**Diclofenac sodium**	0.8
**IA**	3.6
**Cu-IA**	3.4
**Ni-IA**	2.5
**CO-IA**	1.6
**Gd-IA**	2.4
**Sm-IA**	1.9
